# Persistence of self-reactive CD8+ T cells in the CNS requires TOX-dependent chromatin remodeling

**DOI:** 10.1038/s41467-021-21109-3

**Published:** 2021-02-12

**Authors:** Nicolas Page, Sylvain Lemeille, Ilena Vincenti, Bogna Klimek, Alexandre Mariotte, Ingrid Wagner, Giovanni Di Liberto, Jonathan Kaye, Doron Merkler

**Affiliations:** 1 Department of Pathology and Immunology, University of Geneva, Geneva, Switzerland; 2 Research Division of Immunology, Departments of Biomedical Sciences and Medicine, Samuel Oschin Comprehensive Cancer Institute, Cedars-Sinai Medical Center, Los Angeles, CA USA; 3 Division of Clinical Pathology, Geneva University Hospital, Geneva, Switzerland

**Keywords:** Autoimmunity, CD8-positive T cells, Neuroimmunology

## Abstract

Self-reactive CD8^+^ T cells are important mediators of progressive tissue damage in autoimmune diseases, but the molecular program underlying these cells’ functional adaptation is unclear. Here we characterize the transcriptional and epigenetic landscape of self-reactive CD8^+^ T cells in a mouse model of protracted central nervous system (CNS) autoimmunity and compare it to populations of CNS-resident memory CD8^+^ T cells emerging from acute viral infection. We find that autoimmune CD8^+^ T cells persisting at sites of self-antigen exhibit characteristic transcriptional regulation together with distinct epigenetic remodeling. This self-reactive CD8^+^ T cell fate depends on the transcriptional regulation by the DNA-binding HMG-box protein TOX which remodels more than 400 genomic regions including loci such as *Tcf7*, which is central to stemness of CD8^+^ T cells. Continuous exposure to CNS self-antigen sustains TOX levels in self-reactive CD8^+^ T cells, whereas genetic ablation of TOX in CD8^+^ T cells results in shortened persistence of self-reactive CD8^+^ T cells in the inflamed CNS. Our study establishes and characterizes the genetic differentiation program enabling chronic T cell-driven immunopathology in CNS autoimmunity.

## Abstract (web summary)

The transcriptional adaptation processes of harmful self-reactive CD8+ T cells in the central nervous system are not well understood. Here the authors use a system in which self-reactive and virally generated CD8+ T cells are directly compared in vivo and demonstrate that TOX expression contributes to maintenance of auto-reactive CD8+ T cells through alteration of chromatin accessibility.

## Introduction

CD8+ T cells are key players in pathogen elimination and antitumoral immune responses. Conversely, they promote tissue destruction in the context of various autoimmune disorders including multiple sclerosis (MS^1^), type 1 diabetes (T1D^2^), polymyositis^3^, and Hashimoto thyroiditis^4^. Once CD8+ T cells have infiltrated the target organ, they adapt their phenotype and undergo functional reprogramming which is governed by tissue and inflammatory cues^5^. Furthermore, the failure of peripheral suppression by regulatory T cells (Tregs) or the inadequate response of autoreactive CD8+ T cells to environmental inhibitory cues can unleash their tissue-destructive function^6–8^. However, what governs the functional adaptation of autoreactive CD8+ T cells in the inflamed organ remains incompletely understood. With regard to the neuroinflammatory context, we showed that brain infiltrating autoreactive CD8+ T cells trigger an autoimmune disease of the central nervous system (CNS) which depends on T-cell-intrinsic expression of the DNA-binding factor thymocyte selection-associated high mobility group box protein (TOX)^9^. While TOX was originally described as being required for development of T cells of the CD4+ lineage^10^ and innate lymphoid cells^11^, it was recently suggested that this transcription factor (TF) drives a dysfunctional differentiation program of CD8+ T cells in chronic viral infection and cancer^12–17^, which is referred to as exhaustion^18,19^. This adaptation program relies on chronic T-cell receptor (TCR) stimulation and is associated with changes in both the epigenetic and transcriptional landscape^13^. Specifically, T cells with an exhausted phenotype, displaying an increased expression of multiple inhibitory receptors such as PD-1, have been characterized to exhibit a progressive loss of effector functions (e.g., capacity to produce multiple cytokines) in chronic viral infection and cancer. As a result, T cells appear impaired in their ability to mediate defense against a persisting virus or tumor but still retain residual effector functions^20–22^, which can be further reinvigorated by immune checkpoint inhibitors^23^.

Beside TOX, the functional adaptation of T cells is governed by other transcriptional regulators^24^. Among them, TCF-1 marks a subset of chronically stimulated CD8+ T cells which retain stemness properties, and its expression is required for tumor control notably through checkpoint blockade immunotherapy^25–27^. In addition, a recent study indicated that the longevity of self-reactive CD8+ T cells was dependent on the persistence of a stem-like pool of beta cell-specific CD8+ T cells in secondary lymphoid organs of T1D patients^28^. However, the molecular reprogramming of organ infiltrating CD8+ T cells retaining effector function in autoimmune disease context remains poorly explored.

In this work, we harness an experimental autoimmune model of chronic CNS inflammation and show that long-lived self-reactive T cells differentiate by fine-tuning their chromatin accessibility and acquiring a gene expression program that is mediated by TOX. Changes in the epigenetic and transcriptional landscape are associated with phenotypic adaptations of self-reactive CD8+ T cells. Such TOX-dependent cellular reprogramming enables autoimmune CD8+ T cells to persist in the chronically inflamed CNS through a sustained pool of progenitor-like TCF-1^hi^ cells. Our study thus shapes the view of how autoreactive T cells can adapt to chronic TCR stimulation while maintaining pathogenic activity during CNS autoimmunity with potential implications for other chronic immune-driven diseases.

## Results

### Chromatin accessibility changes in self-reactive CD8+ T cells

**Fig. 1:**
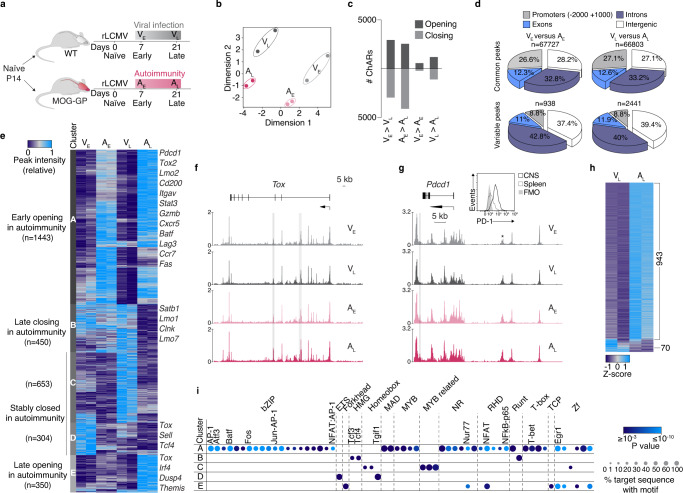
Chromatin accessibility changes in self-reactive CD8+ T cells. 10^4^ naive P14 cells were adoptively transferred into WT and MOG-GP mice. One day later (day 0), mice were challenged i.c. with 10^4^ PFU rLCMV-GP33. Brain infiltrating P14 cells were submitted to ATAC-seq 7 and 21 days after i.c. infection. **a** Experimental scheme. V_E_ and V_L_ (viral infection early and late) correspond to brain infiltrating P14 cells isolated from WT mice (gray) at early (day 7) or late time point (day 21) after i.c. infection. A_E_ and A_L_ (autoimmunity early and late) correspond to brain infiltrating P14 cells isolated from MOG-GP mice (pink) at early (day 7) or late time point (day 21) after i.c. infection. **b** Multidimensional scaling (MDS) plot of chromatin accessibility from A_E_, V_E_, A_L_, and V_L_ P14 cells. Similarity of chromatin accessibility is proportional to the distance between samples. **c** Number of differentially accessible ChARs in each different comparison (Log_2_ FC ≥1; FDR < 0.05). **d** Pie charts showing the distribution for common and variably accessible peaks within promoters, exons, introns, and intergenic regions in the comparisons (V_E_ vs. A_E_) and (V_L_ vs. A_L_). Variable peaks: (Log_2_ FC ≥1; FDR < 0.05). **e** Heatmap of the normalized peak intensity for ChARs displaying differential accessibility in at least one of the comparisons (V_E_ vs. A_E_) or (V_L_ vs. A_L_) (Log_2_ FC ≥1; FDR < 0.05). Hierarchical clustering indicates grouping of samples by ChARs behavior during CNS autoimmunity. Key genes proximal to loci with differential accessibility are indicated for each cluster. Each column represents a biological replicate. **f** ATAC-seq track of *Tox* locus for V_E_, V_L_, A_E_, and A_L_. Differentially accessible ChARs (FDR < 0.05) are highlighted in gray. **g** ATAC-seq tracks of *Pdcd1* locus for V_E_, V_L_, A_E_, and A_L_. Asterisk corresponds to the exhaustion-specific enhancer at −23.8 kb of the TSS. Differentially accessible ChAR (FDR < 0.05) is highlighted in gray. Representative flow cytometry histogram of PD-1 expression in splenic and brain infiltrating P14 cells isolate from WT mice after rLCMV-GP33 i.c. infection (day 21). **h** ATAC-seq Z-score of significantly differentially accessible ChARs (FDR < 0.05) at exhaustion-associated regions^33^. **i** Enrichment of all known transcription factor (TF) motifs within each cluster of differentially accessible ChARs as defined in (**e**). Color depicts the significance of motif enrichment (hypergeometric test) and circle size indicates the fraction of sequence containing a specific motif. All motifs with an enrichment p-value below 10^−3^ in at least one cluster are shown. Source data are provided as a Source data file.

We first assessed chromatin remodeling in brain infiltrating CD8+ T cells under autoimmune conditions as compared to those following acute infection of the CNS. For this purpose, we adoptively transferred congenically marked P14 cells (TCR transgenic CD8+ T cells specific for the Db-restricted immunodominant epitope GP_33-41_ of the glycoprotein (GP) of lymphocytic choriomeningitis virus (LCMV)) into wild-type C57BL/6 mice (WT) or into mice that express the cognate epitope of P14 cells as a neo-self-antigen in myelin-forming cells of the CNS (MOG-GP)^9^. To activate P14 cells, both cohorts were intracranially (i.c.) infected 1 day later with a recombinant and attenuated variant of LCMV (referred to as “rLCMV-GP33”) in which the LCMV GP was replaced by the vesicular stomatitis virus (VSV) GP fused to the leader sequence of LCMV GP including the Db-restricted GP_33-41_ epitope (Fig. 1a). Following rLCMV-GP33 infection, WT mice transiently developed mild clinical symptoms of choriomeningitis, from which they fully recovered within 10 days (Supplementary Fig. 1a). In contrast, MOG-GP mice developed ascending hind limb paresis and ataxia which persisted during the entire observation period of 4 weeks (Supplementary Fig. 1a), while virus was as rapidly cleared as in WT mice within 10 days after i.c. infection (Supplementary Fig. 1b, c). To map the landscape of chromatin accessibility changes that distinguish T cells under autoimmune conditions (MOG-GP) from those in transient viral CNS infection (WT), we performed an assay for transposase-accessible chromatin coupled with high throughout sequencing (ATAC-seq). We sorted brain infiltrating P14 cells from WT and MOG-GP mice both at early time point (day 7; WT mice: V_E_; MOG-GP mice: A_E_) and also at late time point (day 21; WT mice: V_L_; MOG-GP: A_L_) after infection (Fig. 1a). Multidimensional scaling (MDS) of differential chromatin accessible regions (ChARs) across the different samples segregated A_E_, A_L_, V_E_, and V_L_ in four distinct clusters (Fig. 1b). EdgeR analysis (differential expression analysis of digital gene expression data^29^) revealed that in both WT and MOG-GP mice most of the chromatin remodeling took place in CD8+ T cells that underwent differentiation from day 7 to day 21 (Log_2_ fold change (FC) ≥1; false discovery rate (FDR) < 0.05) (Fig. 1c and Supplementary data 1: Differential chromatin accessibility in V_E_, A_E_, V_L_, and A_L_), with an equal number of ChARs that gained and lost accessibility, respectively (Supplementary Fig. 1d). Furthermore, when performing pairwise comparisons in WT and MOG-GP mice, we found more regions remodeled in V_L_ vs. A_L_ than in V_E_ vs. A_E_ (Fig. 1c). Furthermore, ChARs displaying differential openness were enriched in intronic and intergenic regions in effector or memory T cells in autoimmunity (Fig. 1d), potentially reflecting enhancer regulatory regions. Likewise, similar findings were made during the transition from day 7 to day 21 (Supplementary Fig. 1e).

We next performed unsupervised clustering at early (day 7) and late (day 21) time points in order to partition regions whose accessibility diverged during CD8+ T-cell differentiation in the CNS autoimmune context over time (Fig. 1e). This analysis identified five distinct K-mean modules selected visually as the optimal lowest number of clusters reporting correctly the overall variability of ChARs. The following trends of accessibility were observed: two modules of regions remained closed at both time points during autoimmunity (module C and D), two modules demarcated early and late opening (module A and E), and one module corresponded to late closing arising during autoimmunity (module B). The analysis of genes adjacent to ChARs within module A (early opening in autoimmunity) identified the genes encoding for the inhibitory receptors PD-1 and LAG-3, as well as the TFs BATF and TOX2, four previously described exhaustion-associated loci that are epigenetically remodeled^15,30^ (Fig. 1e, Supplementary Fig. 1f and Supplementary data 2: ATAC-seq clustering of V_E_, A_E_, V_L_, and A_L_). Moreover, the module E of greater accessibility occurring at day 21 post infection contains ChARs adjacent to the genes encoding for IRF4 and TOX, two well-known TFs driving T-cell exhaustion^13,31^ (Fig. 1e, f). The distal enhancer region located at −23.8 kb of the transcriptional start site (TSS) of *Pdcd1* showed the same openness in V_L_ and A_L_ supporting the notion that PD-1 maintenance in CNS-resident memory T cells is antigen-independent^32^ (Fig. 1g). To assess whether A_L_ showed epigenetic features related to the T-cell exhaustion program, we evaluated chromatin accessibility within previously described exhaustion-associated ChARs^33^. We found that most of the exhaustion-associated ChARs showed an increased chromatin accessibility in A_L_ as compared to V_L_ (943 out of 1013 ChARs) (Fig. 1h). To determine which TF networks account for the specific differentiation states of CD8+ T cells during CNS autoimmunity, we tested within each module the presence of TF binding motifs with HOMER (Fig. 1i and Supplementary data 3: TF binding motifs identification in clusters identified in Fig. 1e). ChARs opening early during autoimmunity (module A) were enriched for various motifs including bZIP, MYB, NR, RHD, T-box, and Zf TF members. Consistent with the partnerless function of NFAT in driving T-cell exhaustion^34^, we observed a strong enrichment for NFAT motifs without AP-1 in the ChARs that gained accessibility at a late time point in A_L_ as compared to the V_L_ counterpart (module E). Altogether, these data indicate that autoimmune CD8+ T cells acquire an epigenetic landscape reminiscent of exhaustion, which differentiates them from CD8+ T cells persisting in the CNS after transient viral infection.

### Self-reactive CD8+ T cells express a gene program of T-cell exhaustion

**Fig. 2:**
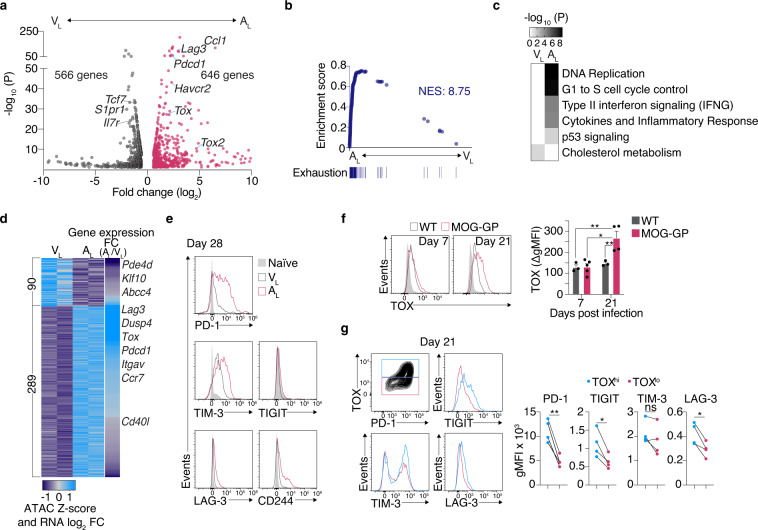
Self-reactive CD8+ T cells express a gene program of T-cell exhaustion. 10^4^ naive P14 cells were adoptively transferred into WT and MOG-GP mice. One day later (day 0), mice were challenged i.c. with 10^4^ PFU rLCMV-GP33. Brain infiltrating P14 cells were FACS sorted for RNA-seq (**a**–**d**) and ATAC-seq (**d**) 21 days later or isolated for flow cytometric analysis at indicated days post infection (**e**–**g**). **a** Volcano plot of differentially expressed genes (DEGs) (FC ≥ 1.5; FDR < 0.05) in A_L_ vs. V_L_ (*n* = 3 mice/group). **b** Gene set enrichment analysis (GSEA) of a signature of exhaustion (chronic LCMV clone 13 infection^24^) in a ranked list of genes differentially expressed by A_L_ vs. V_L_. NES: normalized enrichment score. **c** Heatmap of the significantly enriched WikiPathways for DEGs identified in (**a**). **d** Heatmap illustrating ATAC-seq Z-score and the RNA-seq Log_2_ FC of genes (FDR < 0.05) found adjacent to differentially accessible ChARs (Log_2_ FC ≥1; FDR < 0.05). **e** Representative flow cytometry histograms of inhibitory receptor expression in A_L_ and V_L_ P14 cells 28 days post i.c. rLCMV-GP33 infection. **f** TOX protein expression in P14 cells at day 7 and 21 following i.c. rLCMV-GP33 infection in WT and MOG-GP mice. Representative flow cytometry histograms (left) and summary data (right) (WT mice at day 7, *n* = 3, *p* = 0.0043; WT mice at day 21, *n* = 3, *p* = 0.0113; MOG-GP at day 7, *n* = 5; *p* = 0.0018; MOG-GP mice at day 21, *n* = 4). Light gray histograms indicate the control staining obtained with *Tox*^−/−^ P14 cells. Flow cytometry data are expressed as background-corrected gMFI (ΔgMFI). ΔgMFI values were obtained after subtraction of gMFI values derived from *Tox*^−/−^ P14 cells. Bars represent mean ± SEM. gMFI: geometric mean fluorescence intensity. **g** Stratification of inhibitory receptor expression in TOX^hi^ (blue histograms) and TOX^lo^ (red histograms) A_L_ P14 cells 21 days following i.c. rLCMV-GP33 infection. Flow cytometric analysis of TOX vs. PD-1 expression, inhibitory receptors expression (left) and summary data (right) (*n* = 4 mice/group; PD-1 (*p* = 0.0049); TIGIT (*p* = 0.0204); TIM-3 (*p* = 0.1748); LAG-3 (*p* = 0.0192). ns, not significant; *p ≤ 0.05; **p ≤ 0.01 (one-way ANOVA with Tukey’s post-test for **f**, two-tailed paired *t* test for **g**). Data are representative of at least 2 independent experiments (**e**–**g**). Source data are provided as a Source data file.

We next tested whether the epigenetic profile of A_L_ manifests through a distinct transcriptional state. RNA-seq analyses of brain infiltrating A_L_ and V_L_ revealed 1212 differentially expressed genes (DEGs) (FC ≥ 1.5; FDR < 0.05), including *Tox*, *Tox2*, and various genes encoding for checkpoint molecules (*Pdcd1*, *Havcr2*, *Lag3*) (Fig. 2a and Supplementary data 4: DEGs in V_L_ vs. A_L_). Gene set enrichment analysis (GSEA) further confirmed a signature of exhaustion which is acquired in A_L_ (Fig. 2b). WikiPathways analysis of DEGs further indicated that A_L_ was mainly enriched with functions related to cytokine production and cell division, whereas V_L_ was rather defined by an increased cholesterol metabolism activity, consistent with the role of this pathway for memory T-cell survival^35^ (Fig. 2c). In addition, cross-referencing gene expression with core signatures of known memory T-cell subsets^36^ revealed that both V_L_ and A_L_ were most compatible with tissue-resident memory T cells (Supplementary Fig. 2a).

We then tested the relationship of chromatin accessibility to gene activity by correlating ChARs and related adjacent gene expression in A_L_ and V_L_. This analysis revealed that for a majority of ChAR-gene pairs, openness of the chromatin correlated with gene activity and that key exhaustion DEGs such as *Tox*, *Pdcd1*, and *Lag3* had more accessible loci in A_L_ as compared to V_L_ (Fig. 2d and Supplementary data 5: ATAC-seq Z-score vs. RNA expression). Exhausted CD8+ T cells are characterized by a loss of effector cytokine production and co-expression of various inhibitory receptors^19^. In line with this notion, we observed that A_L_ had a reduced ability to degranulate and co-produce IFN-γ and TNF, which was paralleled by an increased expression of multiple inhibitory receptors such as PD-1, TIM-3, CD244, LAG-3, and TIGIT (Fig. 2e and Supplementary Fig. 2b–d). Similarly, P14 T cells expressed more TOX in MOG-GP than WT mice at 21 days after i.c. infection which was associated with an elevated expression of PD-1, TIGIT, LAG-3 but not TIM-3 (Fig. 2f, g). Finally, central memory P14 cells isolated from the spleen of rLCMV-GP33-infected WT mice retained the potential to acquire TOX and PD-1 when transferred into MOG-GP recipient mice (Supplementary Fig. 2e). Altogether, this suggested that autoimmune CD8+ T cells induce TOX in the CNS and acquire a gene program that distinguishes them from memory T cells that form after transient viral infection.

### TOX is required for self-reactive CD8+ T-cell persistence

**Fig. 3:**
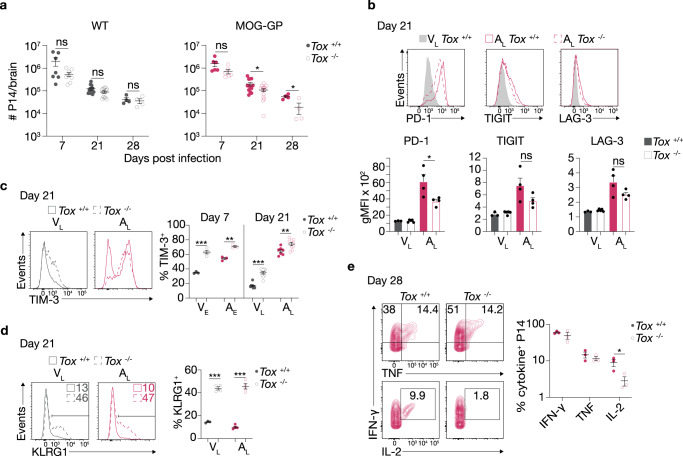
TOX is required for self-reactive CD8+ T-cell persistence. 10^4^ naive *Tox*^+/+^ or *Tox*^−/−^ P14 cells were adoptively transferred into WT and MOG-GP mice. One day later (day 0), mice were challenged i.c. with 10^4^ PFU rLCMV-GP33. Brain infiltrating P14 cells were isolated for flow cytometric analysis at indicated days post infection (**a**–**e**). **a** Flow cytometric enumeration of CNS infiltrating P14 cells at days 7 (*Tox*^+/+^ P14 cells in WT mice, *n* = 7, *Tox*^−/−^ P14 cells in WT mice, *n* = 8, *p* = 0.0739; *Tox*^+/+^ P14 cells in MOG-GP mice, *n* = 6, *Tox*^−/−^ P14 cells in MOG-GP mice, *n* = 6, *p* = 0.0822), 21 (*Tox*^+/+^ P14 cells in WT mice, *n* = 13, *Tox*^−/−^ P14 cells in WT mice, *n* = 14, *p* = 0.0618; *Tox*^+/+^ P14 cells in MOG-GP mice, *n* = 13, *Tox*^−/−^ P14 cells in MOG-GP mice, *n* = 14, *p* = 0.0210), and 28 (*Tox*^+/+^ P14 cells in WT mice, *n* = 4, *Tox*^−/−^ P14 cells in WT mice, *n* = 4, *p* = 0.6420; *Tox*^+/+^ P14 cells in MOG-GP mice, *n* = 4, *Tox*^−/−^ P14 cells in MOG-GP mice, *n* = 4, *p* = 0.0217) post infection. **b** Flow cytometric analysis of inhibitory receptor expression in *Tox*^+/+^ V_L_ (*n* = 3 mice), *Tox*^−/−^ V_L_ (*n* = 5 mice), *Tox*^+/+^ A_L_ (*n* = 4 mice), and *Tox*^−/−^ A_L_ (*n* = 4 mice). PD-1, *p* = 0.0345; TIGIT, *p* = 0.1007; LAG-3, *p* = 0.0997. Representative flow cytometry histograms (above) and summary data (below). **c** Frequency of TIM-3-expressing cells in V_E_ (*Tox*^+/+^, *n* = 3; *Tox*^−/−^, *n* = 4; *p* = 0.0002), A_E_ (*Tox*^+/+^, *n* = 3; *Tox*^−/−^, *n* = 3; *p* = 0.0013), V_L_ (*Tox*^+/+^, *n* = 8; *Tox*^−/−^, *n* = 10; *p* < 0.0001), and A_L_ (*Tox*^+/+^, *n* = 7; *Tox*^−/−^, *n* = 9; *p* = 0.0079). Representative flow cytometry histograms (left) and summary data (right). **d** Frequency of KLRG1-expressing cells in V_L_ (*Tox*^+/+^, *n* = 3; *Tox*^−/−^, *n* = 4; *p* < 0.0001) and A_L_ (*Tox*^+/+^, *n* = 4; *Tox*^−/−^, *n* = 4; *p* < 0.0001). Representative flow cytometry histograms (left) and summary data (right). **e** Intracellular staining for IFN-γ (*p* = 0.3681), TNF (*p* = 0.374), and IL-2 (*p* = 0.0436) in *Tox*^+/+^ and *Tox*^−^^/−^ A_L_ cells at day 28 post infection after in vitro stimulation with KAVYNFATC peptide. Numbers indicate the frequency of cytokine-producing cells within each quadrant. Representative flow cytometry plots (left) and summary data (right) (*n* = 3 mice/group). ns, not significant; *p ≤ 0.05; **p ≤ 0.01; ***p ≤ 0.001 (two-tailed unpaired *t* test for **a**–**e**). Data represent the pool of two independent experiments (**a** and **c**) or are representative of at least two independent experiments (**b**, **d**, and **e**). Bars and horizontal lines represent mean ± SEM. Source data are provided as a Source data file.

We next determined the differential contribution of TOX for T-cell differentiation in acute infection and in CNS autoimmunity. Here, we transferred naive *Tox* competent (*Tox*^+/+^) or deficient (*Tox*^−/−^^10^) P14 cells into WT or MOG-GP mice one day prior to i.c. infection with rLCMV-GP33. *Tox*^−/−^ P14 cells expanded and contracted similarly to *Tox*^+/+^ P14 cells counterparts in the brain of WT mice (Fig. 3a). While the initial expansion in this setting was not affected by the absence of *Tox* in the brain of MOG-GP mice*, Tox*^−/−^ P14 cells underwent a more rapid culling in the autoimmune context. The difference started to be significant as of day 21 when the level of TOX was higher in MOG-GP as compared to WT mice (see Fig. 2f). To compare CD8+ T-cell responses within the same host and thus tissue microenvironment, we performed co-transfer of naive *Tox*^*−/−*^ and *Tox*^*+/+*^ P14 cells into WT and MOG-GP mice, one day prior to i.c. infection with rLCMV-GP33 (Supplementary Fig. 3a). In this competitive setting, primary expansion of *Tox*^−/−^ and *Tox*^+/+^ P14 cells in the blood was similar in WT and MOG-GP mice (Supplementary Fig. 3b). Compared to *Tox*^+/+^, however, the representation of *Tox*^−/−^ P14 cells in the CNS was already reduced 7 days after infection in both cohorts (Supplementary Fig. 3b). In addition, we observed that between 21 to 35 days after infection (contraction-memory phase) the ratio of *Tox*^−/−^ to *Tox*^+/+^ P14 cells in the CNS continued to drop over time in MOG-GP recipient whereas it remained stable in WT recipient mice (Supplementary Fig. 3c).

Altogether, these findings indicate that in a competitive setting, deficiency of *Tox* does not affect the initial expansion of CD8+ T cells but results in an intrinsic disadvantage for the recruitment or survival of CD8+ T cell infiltrates into the CNS, and additionally in the autoimmune condition, the persistence of these cells in the CNS.

We furthermore investigated inhibitory receptor expression on CD8+ T cells in the CNS. Among the different inhibitory receptors investigated, we found in the single transfer experiment a lower expression of PD-1, and likewise a trend for reduced TIGIT and LAG-3 expression in the absence of TOX (Fig. 3b). However, the frequency of TIM-3+ cells, which predominantly co-expressed the tissue residency marker CD103, was increased in *Tox*^−/−^ P14 cells (Fig. 3c and Supplementary Fig. 3d). In addition, the TOX-dependent CD8+ T-cell intrinsic effect on PD-1 and TIM-3 expression was confirmed in the competitive co-transfer setting (Supplementary Fig. 3e).

We further found a higher frequency of *Tox*^−/−^ P14 cells expressing the short-lived effector T-cell marker KLRG1 and granzyme B (GzmB) in both acute infection and CNS autoimmunity (Fig. 3d, Supplementary Fig. 3f). Despite a more terminal effector phenotype, *Tox*^−/−^ P14 produced an equal amount of IFN-γ and TNF (Fig. 3e). In addition, IL-2 production, which marks the stemness properties of CD8+ T cells^37^, was significantly reduced in absence of TOX (Fig. 3e).

Collectively, these results suggest that TOX acts as T-cell intrinsic regulator on two uncoupled axes. First, TOX provides an advantage for CD8+ T-cell persistence under CNS autoimmune conditions. Second, it restrains terminal effector differentiation in both acute infection and CNS autoimmunity.

### TOX epigenetically reprograms self-reactive CD8+ T cells

**Fig. 4:**
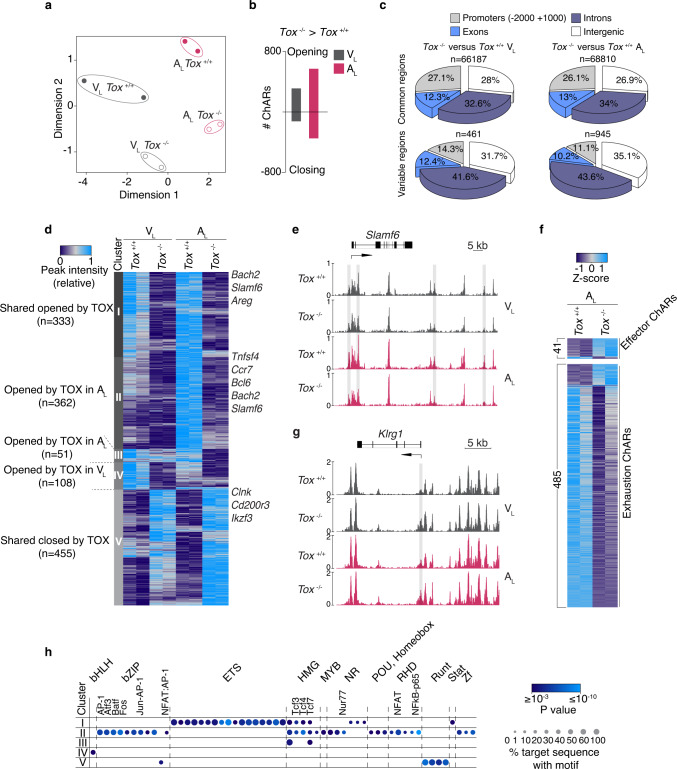
TOX epigenetically reprograms self-reactive CD8+ T cells. 10^4^ naive *Tox*^+/+^ or *Tox*^−/−^ P14 cells were adoptively transferred into WT and MOG-GP mice. One day later (day 0), mice were challenged i.c. with 10^4^ PFU rLCMV-GP33. Brain infiltrating P14 cells were FACS sorted and submitted to ATAC-seq 21 days later. **a** MDS plot of chromatin accessibility from *Tox*^+/+^ or *Tox*^−/−^ V_L_ and A_L_ cells. Similarity of chromatin accessibility is proportional to the distance between samples. **b** Number of differentially accessible ChARs in *Tox*^+/+^ vs. *Tox*^−/−^ V_L_ and A_L_ cells (Log_2_ FC ≥1; FDR < 0.05). **c** Pie charts showing the distribution for common and variably accessible peaks within promoters, exons, introns and intergenic regions in *Tox*^+/+^ vs. *Tox*^−/−^ V_L_ and A_L_ comparisons. Variable peaks: (Log_2_ FC ≥1; FDR < 0.05). **d** Heatmap of the normalized peak intensity for ChARs displaying differential accessibility in at least one comparison (*Tox*^+/+^ vs. *Tox*^−/−^ V_L_) or (*Tox*^+/+^ vs. *Tox*^−/−^ A_L_) (Log_2_ FC ≥1; FDR < 0.05). Hierarchical clustering indicates grouping of samples by TOX-dependent ChAR behavior. Key genes proximal to loci with differential accessibility are indicated for each cluster. Each column represents a biological replicate. **e** ATAC-seq track of *Slamf6* locus for *Tox*^+/+^ and *Tox*^−/−^ V_L_ and A_L_ cells. Differentially accessible ChARs (FDR ≤0.05) are highlighted in gray. **f** ATAC-seq Z-score of significantly differentially accessible ChARs identified in the comparison *Tox*^+/+^ vs. *Tox*^−/−^ A_L_ (FDR < 0.05) at effector and exhaustion-associated regions^33^. **g** ATAC-seq track of *Klrg1* locus for *Tox*^+/+^ and *Tox*^−/−^ V_L_ and A_L_ cells. Differentially accessible ChARs (FDR < 0.05) are highlighted in gray. **h** Enrichment of all known TF motifs within each cluster of differentially accessible ChARs as defined in (**d**). Color depicts the significance of motif enrichment (hypergeometric test) and circle size indicates the fraction of sequence containing a specific motif. All motifs with an enrichment p-value below 10^−3^ in at least one cluster are shown. Source data are provided as a Source data file.

To investigate whether the observed phenotype of A_L_ cells was driven by TOX-dependent chromatin accessibility changes, we performed ATAC-seq profiling from *Tox*^−/−^ and *Tox*^+/+^ in A_L_ and V_L_. MDS plot of genome-wide variability of chromatin accessibility across the different samples indicated that TOX controls the openness of ChARs in both A_L_ and V_L_ (Fig. 4a). In line with the higher expression of TOX in A_L_ than in V_L_, we found a majority of differentially accessible ChARs in MOG-GP (945 in A_L_ vs. 461 in V_L_) and among them, 583 were more opened and 362 regions were more closed in presence of TOX (Fig. 4b). Almost 80% of the TOX-dependent chromatin remodeling occurred in intronic and intergenic regions while only 10% were at promoter regions (Fig. 4c). To assess the relative contribution of TOX as chromatin modifier in CNS autoimmunity or after acutely resolved infection, we plotted the chromatin accessibility of ChARs that varied in absence of TOX in A_L_ and V_L_ (Log_2_ FC ≥ 1; FDR < 0.05) (Fig. 4d and Supplementary data 6: TOX-dependent chromatin accessibility changes in A_L_ and V_L_). Unsupervised clustering analysis identified five distinct modules of TOX-dependent epigenetic changes based on whether they were unique to CNS autoimmunity, unique to acute infection or equally found in the two settings. In module I and V of ChARs, *Tox*^−/−^ P14 cells followed a similar epigenetic trajectory in both WT and MOG-GP recipient mice (Fig. 4d and Supplementary data 7: ATAC-seq clustering of *Tox*^−/−^ and *Tox*^+/+^ V_L_ and A_L_). In module IV, 108 regions got preferentially remodeled through TOX in V_L_ (Fig. 4d). In agreement with the higher expression of TOX in A_L_, TOX preferentially imposed the opening of 362 ChARs in module II and 51 ChARs in module III during CNS autoimmunity (Fig. 4d). Proximal to ChARs that were closing in absence of TOX, there were key genes associated with memory and exhaustion commitment including *Ccr7*, *Bcl6*, *Bach2*, and *Slamf6* (Fig. 4d, e). Consistent with the role of CLNK in sensing IL-2^38^ and TOX-dependent IL-2 production in T cells (see Fig. 3e), we found TOX-dependent epigenetic changes adjacent to *Clnk* in module V (Fig. 4d).

While most of the differentially accessible ChARs associated with terminal effector differentiation opened in absence of TOX, the exhaustion-specific loci were predominantly closing in *Tox*^−/−^ A_L_ (Fig. 4f). Statistically significant differentially accessible ChARs could also be identified in key genes including *Klrg1* and *Tcf7*, which gained and lost, respectively, accessibility in absence of TOX (Fig. 4g and Supplementary Fig. 4a). TF motif enrichment analysis with HOMER confirmed the depletion of HMG-box motifs including Tcf3, Tcf4, and Tcf7 in *Tox*^−/−^ P14 in module I, II, and to a lesser extent in module III, suggesting that TOX influences direct or indirect TCF-1-related epigenetic events during autoimmunity (Fig. 4h and Supplementary data 8: TF binding motifs identification in clusters identified in Fig. 4d). This analysis further revealed an over-representation of binding sites for RUNX and E26 transformation-specific (ETS) factors in ChARs that were, respectively, opening and closing in absence of TOX in WT and MOG-GP mice. Moreover, ChARs which displayed a decreased accessibility primarily in *Tox*^−/−^ A_L_ cells were enriched in binding sites for AP-1, NFAT, and NFAT: AP-1 (Fig. 4h) suggesting that TOX epigenetically reprograms A_L_ cells by modulating accessibility of this distinct set of TFs during CNS autoimmunity. Collectively, these data indicate that TOX acts as a major chromatin modifier which adapts its epigenetic remodeling activity according to the context in which CD8+ T cells are arising.

### TOX imposes a gene program of exhaustion in self-reactive CD8+ T cells

**Fig. 5:**
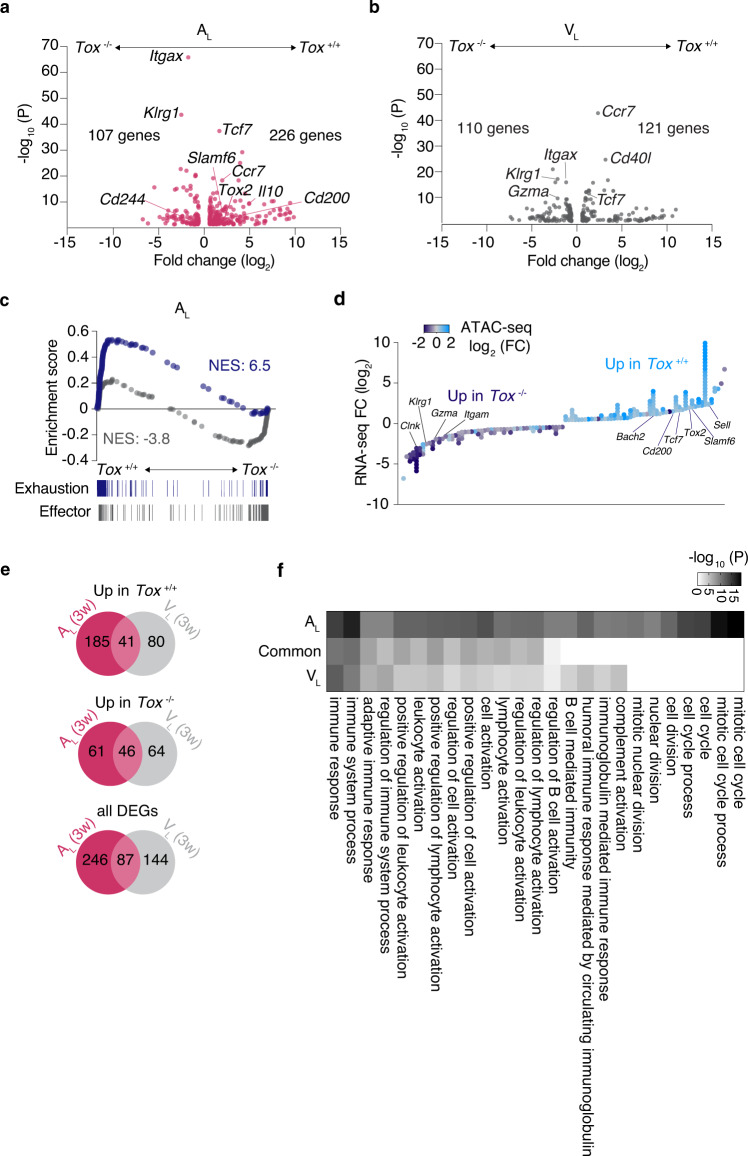
TOX imposes a gene program of exhaustion in self-reactive CD8+ T cells. 10^4^ naive *Tox*^+/+^ or *Tox*^−/−^ P14 cells were adoptively transferred into MOG-GP (**a**, **c**–**f**) and WT mice (**b**, **e**, **f**). One day later (day 0), mice were challenged i.c. with 10^4^ PFU rLCMV-GP33. Brain infiltrating P14 cells were FACS sorted for RNA-seq (**a**–**f**) and ATAC-seq (**d**) 21 days later. **a** Volcano plot of differentially expressed genes (DEGs) (FC ≥1.5; FDR ≤0.05) in *Tox*^+/+^ vs. *Tox*^−/−^ A_L_ cells (*n* = 3 mice/group). **b** Volcano plot of differentially expressed genes (DEGs) (FC ≥1.5; FDR ≤0.05) in *Tox*^+/+^ vs. *Tox*^−/−^ V_L_ cells (*n* = 3 mice/group). **c** GSEA of a signature of exhaustion and effector differentiation^33^ in a ranked list of genes differentially expressed by *Tox*^+/+^ vs. *Tox*^−/−^ A_L_ cells. NES: normalized enrichment score. **d** Diamond plot of differentially accessible ChARs adjacent to the top 50 up- and downregulated genes identified in (**a**). Top DEGs which possess at least one differential ChAR within a 15 kb distance are shown. Each diamond represents a ChAR assigned to a gene. Color code depicts the gain or loss of accessibility (Log_2_ FC) in the comparison *Tox*^+/+^ vs. *Tox*^−/−^ A_L_ cells. **e** Venn diagram showing the overlap of DEGs found in the comparisons *Tox*^+/+^ vs. *Tox*^−/−^ in both A_L_ and V_L_ cells. **f** Enrichment of biological processes (GO terms) among the DEGs (up in *Tox*^+/+^ vs. *Tox*^−/−^) commonly or uniquely found in A_L_ and V_L_ cells. The top 10 most significantly enriched GO terms are shown for each category.

To assess whether the observed TOX-dependent chromatin alterations manifest through a distinct transcriptional landscape, we performed RNA-seq in *Tox*^+/+^ and *Tox*^−/−^ A_L_ and V_L_ cells, respectively. Transcriptional analysis of *Tox*^−/−^ and *Tox*^+/+^ P14 cells revealed 333 differentially expressed genes in A_L_ and 231 in V_L_ (FC ≥1.5; FDR < 0.05) (Fig. 5a, b and Supplementary data 9: DEGs in *Tox*^−/−^ vs. *Tox*^+/+^ V_L_ and A_L_). Three weeks after i.c. infection with rLCMV-GP33 and regardless of the nature of the recipient mice, *Tox*^−/−^ A_L_ and V_L_ cells showed evidence of an increased effector phenotype as judged by an increased transcript abundance for genes encoding typical markers of short-lived effector cell differentiation. In contrast, *Tox*^−/−^ A_L_ and V_L_ had a reduced expression of memory T-cell genes such as *Ccr7* and *Tcf7* (Fig. 5a, b). Compared with V_L_, *Tox*^−/−^ A_L_ showed a striking decreased expression of exhaustion-associated genes such as *Tox2* and *Cd200*, the ligand of the inhibitory receptor CD200R^15,39^ (Fig. 5a). Indeed, GSEA confirmed that expression of exhaustion-associated genes^24^ was reduced, while expression of genes associated with short-lived effector cell differentiation was enriched in *Tox*^−/−^ as compared to *Tox*^+/+^ P14 cells (Fig. 5c and Supplementary Fig. 5a). We then tested whether the previously identified TOX-dependent epigenetic changes were paralleled by a differential activity of genes adjacent to detected ChARs. By cross-referencing accessibility and gene expression, we observed a strong correlation between chromatin openness and gene activity suggesting that TOX controls gene expression by interfering with chromatin accessibility at key loci (Fig. 5d). Next, we sought to determine to which extent the TOX-driven transcriptional program overlapped in brain infiltrating CD8+ T cells arising in V_L_ after acute infection and A_L_ during CNS autoimmunity. Overall, 74% (246/333 genes) and 62% (144/231 genes) of the genes responsive to *Tox* disruption were uniquely found in A_L_ and V_L_, respectively (Fig. 5e). In this regard, gene ontology (GO) enrichment analysis of biological processes indicated that TOX-responsive genes in A_L_ selectively overlapped with genes representative of mitotic activity and cell division (Fig. 5f). In contrast, TOX-dependent genes in V_L_ were enriched for more general GO terms linked to the regulation of lymphocyte activation, which was also enriched to some extent in A_L_ (Fig. 5f). Altogether, these data confirmed that TOX can orchestrate a distinct gene program through epigenetic changes depending on the context from which CD8+ T cells arise.

### TOX preserves the pool of self-reactive TCF-1^hi^ CD8+ T cells

**Fig. 6:**
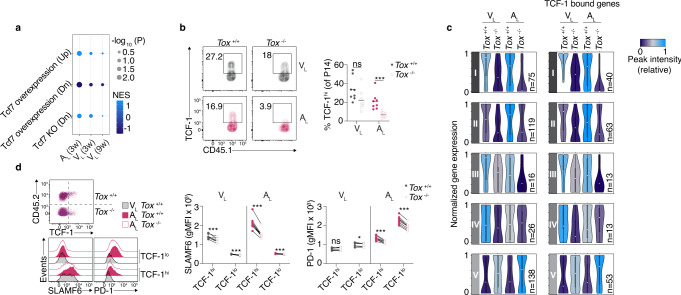
TOX predominantly preserves the pool of self-reactive TCF-1^hi^ CD8+ T cells. 10^4^ naive *Tox*^+/+^ or *Tox*^−/−^ P14 cells were adoptively transferred into WT and MOG-GP mice. One day later (day 0), mice were challenged i.c. with 10^4^ PFU rLCMV-GP33 and brain infiltrating P14 cells were FACS sorted for RNA-seq (**a** and **c**), ATAC-seq (**c**) and flow cytometric analysis (**b**) at indicated days post infection. **a** GSEA of TCF-1 specific signatures^27^ in a ranked list of genes differentially expressed by *Tox*^+/+^ vs. *Tox*^−/−^ A_L_ and V_L_ at indicated time points. NES: normalized enrichment score. **b** Frequency of TCF-1-expressing cells in V_L_ (*Tox*^+/+^, *n* = 8; *Tox*^−/−^, *n* = 9; *p* = 0.0554) and A_L_ (*Tox*^+/+^, *n* = 8; *Tox*^−/−^, *n* = 9; *p* = 0.0004) cells 21 days after infection. Representative flow cytometry plot (left) and summary data (right). Horizontal lines represent the mean. **c** Violin plots illustrating normalized expression of genes found proximal to differentially accessible ChARs (maximum distance to gene = 100 kb) for each module as defined in Fig. 4d. The bounds of the boxes indicate the 25th and 75th percentiles, the center (dot) reflects the median, the lower whisker indicates the minimum, and the upper indicates the maximum of normalized gene expression and violin colors indicate the average peak intensity of ChAR-gene pairs within each module (module I, *n* = 75; module II, *n* = 119; module III, *n* = 16; module IV, *n* = 26; module V, *n* = 138). Genes showing at least one TCF-1 binding event are depicted within each module (module I, *n* = 40; module II, *n* = 63; module III, *n* = 13 module IV, *n* = 13; module V, *n* = 53). **d** Congenically distinct naive *Tox*^+/+^ and *Tox*^−/−^ P14 cells were mixed 1:1 and adoptively transferred ﻿ into WT and MOG-GP mice. One day later (day 0), mice were challenged i.c. with 10^4^ PFU rLCMV-GP33 and brain infiltrating P14 cells were isolated for flow cytometric analysis 21 days later. SLAMF6 and PD-1 expression stratified in TCF-1^hi^ and TCF-1^lo^ subsets in V_L_ (*n* = 7 mice) and A_L_ cells (*n* = 8 mice). Gating strategy and representative flow cytometry histograms (left) and summary data (right). SLAMF6 (V_L_ TCF-1^hi^, *p* = 0.0007; V_L_ TCF-1^lo^, *p* = 0.0001; A_L_ TCF-1^hi^, *p* < 0.0001; A_L_ TCF-1^lo^, *p* < 0.0001); PD-1 (V_L_ TCF-1^hi^, *p* = 0.5355; V_L_ TCF-1^lo^, *p* = 0.0113; A_L_ TCF-1^hi^, *p* < 0.0001; A_L_ TCF-1^lo^, *p* < 0.0001). ns, not significant; *p ≤ 0.05; ***p ≤ 0.001 (two-tailed unpaired *t* test for (**b**) and two-tailed paired *t* test for (**d**)). Data represent the pool of two independent experiments (**b**) or are representative of at least two independent experiments (**d**). Source data are provided as a Source data file.

Our RNA-seq data indicated that expression of the gene encoding for TCF-1 (Tcf7) was nearly ablated in absence of TOX in self-reactive CD8+ T cells and, to a lesser extent, in CD8+ T cells that formed 3 weeks after the resolved infection with rLCMV-GP33 (Supplementary Fig. 6a). TCF-1 marks a subset of exhausted T cells with memory-like functions that keep their stemness and proliferative properties^26^. Consistent with the higher expression of Tcf7 in presence of TOX and the notion that TCF-1 inversely correlates with TIM-3, we found that *Tox*^−/−^ P14 cells displayed an increased expression of TIM-3 (Fig. 3c). This relationship between TOX and Tcf7, however, disappeared 9 weeks after viral infection (Supplementary Fig. 6b and Supplementary data 9: DEGs in *Tox*^−/−^ vs. *Tox*^+/+^ V_L_ and A_L_). In addition, we used GSEA to better understand the role of TOX in controlling TCF-1-related gene signatures^27^ in A_L_ and V_L_ 3 and 9 weeks after infection. This comparative analysis revealed the greatest enrichment of TOX-dependent TCF-1 signature in A_L_ as compared to V_L_ 3 weeks after infection. Of note, no enrichment of TCF-1 signature remained detectable in V_L_ 9 weeks after infection (Fig. 6a). To confirm that protein expression of TCF-1 was affected in the absence of TOX, we performed flow cytometric analysis in *Tox*^−/−^ and *Tox*^+/+^ A_L_ and V_L_. We found that the proportion of TCF-1-expressing cells in *Tox*^−/−^ P14 cells was dramatically decreased in A_L_ (Fig. 6b). In contrast, there was only a slight trend towards a decrease expression of TCF-1 in V_L_ cells that formed after acutely resolved infection in the brain (Fig. 6b). This tendency was reflected by absolute quantification of brain infiltrating T cells which showed that in absence of TOX, the abundance of progenitor-like TCF-1^hi^ subset was predominantly affected in A_L_ (Supplementary Fig. 6c). In contrast, the TCF-1^lo^ subset did not follow the same pattern. Thus, TOX is a critical regulator of TCF-1^hi^ T-cell subset persistence during CNS autoimmunity and to a lesser extent after acute resolved viral infection (Supplementary Fig. 6c).

To elucidate the molecular mechanism by which TOX controls the presence of TCF-1-expressing cells during CNS autoimmunity, we interrogated our ATAC-seq data by evaluating the changes in the expression of genes that were found proximal to regions that became more or less accessible in the absence of TOX (Fig. 6c and Supplementary data 10: Gene expression changes associated with TCF-1 binding in ATAC-seq clusters identified in Fig. 4d). The analysis of ChAR-gene pairs revealed that most of the TOX-dependent epigenetic changes were functionally relevant since the chromatin openness correlated with the gene expression level. Given the role of TCF-1 in coordinating chromatin accessibility changes upon binding^40^, we reasoned that TOX-dependent control of gene expression through chromatin remodeling would mainly affect TCF-1 bound genes. Thus, we interrogated whether these changes in expression concerned genes which show TCF-1 binding events from a previously published TCF-1 ChiP-seq dataset from naive CD8+ T cells^41^. Considering genes with at least one TCF-1 binding event (*n* = 5472), we found that about 50% of the identified genes associated with differential accessible ChARs were TCF-1-bound genes (Fig. 6c and Supplementary data 10: Gene expression changes associated with TCF-1 binding in ATAC-seq clusters identified in Fig. 4d) indicating that TOX expression is required to partly recapitulate the epigenetic and transcriptional landscape invoked by TCF-1. In addition, we found that TOX expression was higher in the memory-like TCF-1^hi^ subset, a trend which was already noticeable 7 days after infection of MOG-GP mice (Supplementary Fig. 6d). Thus, we tested whether the absence of TOX would affect the expression of key genes in the TCF-1^hi^ subset. Our RNA-seq and ATAC-seq data revealed that *Tox*^−/−^ P14 cells had a reduced expression of *Slamf6* which was accompanied by massive changes in chromatin accessibility. Interestingly, SLAMF6 is an inhibitory receptor which is highly co-expressed with TCF-1 in the progenitor-like subset of exhausted CD8+ T cells in cancer^42^. We thus compared the expression of SLAMF6 and PD-1 in TCF-1^hi^ and TCF-1^lo^ subsets, respectively, in *Tox*^+/+^ and *Tox*^−/−^ P14 cells. In contrast to TCF-1^lo^ subset, the absence of TOX profoundly decreased the expression of SLAMF6 in the TCF-1^hi^ subset of A_L_ cells (Fig. 6d), a pattern which was also noticeable, although to a lesser extent, in V_L_ cells. Of note, in absence of TOX the decreased expression of PD-1 was equally observed within the TCF-1^hi^ and TCF-1^lo^ subsets during CNS autoimmunity. Altogether, these data demonstrated that TOX favors the maintenance of the reservoir of autoimmune TCF-1^hi^ cells by invoking a characteristic epigenetic and transcriptional program.

## Discussion

CD8+ T cells show remarkable functional adaptation depending on the context to which they are exposed. While in cancer and infections the molecular underpinnings regulating such T-cell differentiation programs are beginning to be deciphered, our understanding of such processes in chronic autoimmune diseases is still sparse. In particular, it remains unexplored how the phenotype of T cells is regulated and to what extent it influences the course of an established chronic autoimmune disease. We addressed here these questions by following the differentiation fate of self-reactive CD8+ T cells in a model of CNS autoimmunity. Early after autoimmune disease precipitation, we found that self-reactive CD8+ T cells harbored many of the epigenetic traits seen in anti-viral CD8+ T-cell effectors. However, in the further course, CD8+ T cells targeting self-antigens in oligodendrocytes bifurcated from classical anti-viral memory cells by acquiring epigenetic marks characteristics with populations of exhausted CD8+ T cells^33^. Among the various epigenetic changes associated with an exhaustion phenotype in self-reactive CD8+ T cells, we found the gene loci of *Tox* to be remodeled. We previously showed that TOX is instrumental for autoreactive CD8+ T cells to precipitate autoimmune CNS disease^9^. Several groups have meanwhile reported that TOX is a major driver of T-cell exhaustion in chronic infection and cancer^12–16^, while others found it expressed in highly functional effector memory CD8+ T-cell subsets during persistent human viral infection^43^. Our results show that TOX invoked an epigenetic and transcriptional program similar to exhaustion under an autoimmune disease condition. This includes TOX-dependent expression of various inhibitory receptors, such as PD-1 and TIM-3, and profound epigenetic remodeling of exhaustion-associated chromatin regions including *Tox2*^15^. However, we also found that in the absence of TOX, differentiation of self-reactive CD8+ T cells was skewed towards short-lived effector cells that vanished over time from the inflamed CNS. This supports the idea that the fate of self-reactive CD8+ T cells reflects a functional adaptation which enables T cells to cope with chronic TCR stimulation in autoimmune conditions. This is in line with previous reports showing that TOX mediated survival of CD8+ T cells in chronic viral infection and cancer^12–14^. In this regard, we found that some of the TOX-dependent epigenetic changes and accessibility landscape for TFs occur uniquely under autoimmune conditions. In particular, a large proportion of ChARs that gained accessibility as a function of TOX contained motifs for TCF-1 that were co-enriched with bZIP members including AP-1 and NFAT:AP-1 (see Fig. 4d). This suggests that as previously found for chronic^44^ and acute^45^ infections, TOX controls T-cell fate decisions through TCF-1, but during CNS autoimmunity in cooperation with other TFs. Thereby, TOX preserved stemness properties of autoreactive T cells which provided an advantage for these cells to persist in the inflamed CNS and consequently could propagate their tissue-destructive function in chronic autoimmune disease condition^9^.

From an evolutionary point of view, the exhaustion program of T cells seems to be beneficial to avoid excessive tissue damage during inflammatory processes. This concept is supported by the notion that cancer patients that undergo checkpoint blockade immunotherapy can develop autoimmune complications^46^. However, it remains unclear to which extent self-reactive CD8+ T cells displaying an exhausted phenotype can still perpetuate tissue damage in chronic T-cell-mediated autoimmune diseases. While it has been suggested that T-cell exhaustion signature concurs with a lower risk of relapse and symptom recurrence^47^, depletion of PD-1+ T cells in established type I diabetes (T1D) and experimental autoimmune encephalomyelitis (EAE) models favorably influences the clinical outcome^48^. The latter findings suggest that such T cells—despite considered as non- or less-responsive in cancer—can still exert pathogenic functions and contribute to disease progression in autoimmune disease conditions. In addition, it still needs to be investigated to what extent the epigenetic program of self-reactive CD8+ T cells represents a fixed state or can be reversed under certain conditions that eventually manifests itself in the form of disease relapses. Furthermore, it can be speculated that the net tissue damage within the inflamed organ caused by self-reactive CD8+ T cells is the result of a finely tuned balance between T-cell persistence on the one hand and differentiation into short-lived effector CD8+ T cells on the other hand. This is particularly true for the CNS, which is equipped with specific immunosuppressive strategies^49^, aiming at limiting unwanted harmful inflammation that can inflict potential irreversible damage.

In several chronic autoimmune diseases of solid organs, a growing body of evidence suggests that autoreactive T cells residing in the inflamed organ contribute to the tissue-damaging process^50^. Given that such T_RM_ cells are in disequilibrium with the circulation, autoreactive T_RM_ are exposed to continuous TCR stimulation which entails corresponding cellular adaptation. With regard to the CNS, T_RM_-like cells expressing the tissue residency marker CD103 are found within inflammatory MS lesions^51,52^ and clonally expanded CD8+ T cells that bear a T_RM_-like gene signature are enriched within the cerebrospinal fluid of MS patients^53^. In line with this notion, our data suggest that self-reactive CD8+ T cells in the chronically inflamed CNS of MOG-GP mice most likely correspond to T_RM_ cells. The finding that T_RM_-like cells persist in the CNS in a TOX-dependent manner in animals during autoimmunity, together with the previous observation that TOX-deficient self-reactive CD8+ T cells exhibit reduced encephalitogenic properties^9^, suggest that T_RM_-like cells contribute to disease pathogenesis. Accordingly, the ability of T_RM_-like cells (see Fig. 5e) to produce chemokines and pro-inflammatory cytokines indicates that these cells retained a degree of responsiveness that could contribute to tissue damage, although the exact mode of action remains to be elucidated. Our data also suggest that treatment targeting T_RM_ within the inflamed organ could have clinical benefit. The latter may also explain why current therapies, which interfere with the migration of T cells into the CNS, are less effective in progressive MS in which T_RM_-like cells are frequently found^51^.

In summary, our study delineates the epigenetic reprogramming of self-reactive CD8+ T cells in which TOX plays a crucial role. TOX-dependent fate commitment enables self-reactive CD8+ T cells to persist in the CNS by preserving the pool of TCF-1^hi^ progenitor cells in cooperation with distinct TFs. Unraveling the molecular mechanisms modulating the longevity of self-reactive CD8+ T cells in the target organ in chronic autoimmune diseases may thus have implications for therapeutic approaches aiming at restraining destructive immune responses.

## Methods

### Mice

C57BL/6J WT were obtained from Charles River (France). C57BL/6 *Tox*^−/− 10^ were kindly provided by J. Kaye and crossed with C57BL/6J P14 TCR transgenic mice with a different CD45 allele to perform adoptive transfer experiments. C57BL/6J MOG-GP (MOGi^Cre/+^: Stop-GP^flox/+^) mouse line was generated by crossing mice expressing the Cre-recombinase under the control of the oligodendrocyte-specific promoter (C57BL/6J MOGiCre^54^; with C57BL/6J Stop-GP mice^9^). All mice were lodged under specific-pathogen-free P2 conditions in the animal facilities of the University Medical Center of Geneva. Male and female sex and age-matched mice between 6 and 12 weeks of age were used for experiments. All animal experiments were authorized by the cantonal veterinary office of Geneva and performed in agreement with the Swiss law for animal protection.

### Purification of naive P14 cells and adoptive P14 cell transfer

Naive P14 cells from spleen were purified using naive CD8a+ T Cell Isolation Kit (Miltenyi Biotec, 130-104-075) and subsequently separated by AutoMACS (Miltenyi Biotec). Purity of the cells (95%) was confirmed by flow cytometry. 10^4^ P14 cells were adoptively transferred intravenously into WT and MOG-GP mice 1-day prior to viral infection.

### Virus infection

Recombinant LCMV were generated and propagated on baby hamster kidney 21 cells BHK21 (ATCC^®^ CCL-10)^55^. Virus stocks were titrated using MC57G cells (ATCC^®^ CRL-2295) according to established methods^56^. The recombinant rLCMV-GP33 expresses the signal sequence of LCMV glycoprotein (GP) harboring the GP_33-41_ epitope fused to the VSV GP instead of the LCMV full-length GP. For transient virus infection in the brain, 10^4^ plaque-forming units (PFU) of rLCMV-GP33 were diluted in 30 μl of minimum essential medium (Gibco) before intracranial (i.c.) injection. For peripheral challenge, mice were intravenously injected into the tail vein with 10^5^ PFU of rLCMV-GP33. Infected animals were monitored daily for occurrence of classical experimental autoimmune encephalomyelitis (EAE) symptoms and scored as follows. 1, flaccid tail; 2, impaired righting reflex and hind limb weakness; 3, complete hind limb paralysis; 4, complete hind limb paralysis with partial fore limb paralysis; 5, moribund. Severely diseased animals were immediately sacrificed.

### Immunohistochemistry

Brain tissues were collected and fixed in 4% paraformaldehyde (PFA) overnight at 4 °C. Subsequently, tissues were embedded in paraffin and endogenous peroxidases (PBS/3% H_2_O_2_) were neutralized and unspecific binding blocked (PBS/10% FCS) on brain tissue sections (4 μm). After antigen retrieval, sections were stained with a poly‐reactive anti‐LCMV nucleoprotein rabbit serum^57^. Bound primary antibodies were visualized with HRP-based detection with 3,3′-diaminobenzidine (DAB) as chromogen (Dako, K3468; hemalaun counterstaining of nuclei).

### FACS analysis and sorting

The following fluorophore-conjugated antibodies for flow cytometry were purchased from BioLegend (anti-CD8a Brilliant violet 510 (53-6.7), anti-CD8a Brilliant violet 605 (53-6.7), anti-CD45.1 Alexa Fluor 700 (A20), anti-CD45.1 Pacific Blue (A20), anti-CD45.1 Brilliant violet 785 (A20), anti-CD45.2 PE/Cyanine7 (104), anti-CD107a FITC (1D4B), anti-CD244 PerCP/Cyanine5.5 (m2B4 (B6)458.1), anti-granzyme B Alexa Fluor 647 (GB11), anti-IFN-γ APC (XMG1.2), anti-IL-2 PE (JES6-5H4), anti-KLRG1 FITC (2F1), anti-KLRG1 Brilliant Violet 421 (2F1), anti-LAG-3 APC (C9B7W), anti-PD-1 PE (29 F.1A12), anti-PD-1 PerCP/Cyanine5 (29F.1A12), anti-PD-1 FITC (29F.1A12), anti-TIGIT PE/Cyanine7 (1G9), anti-TIM-3 Brilliant violet 605 (RMT3-23), anti-TIM-3 Brilliant violet 711 (RMT3-23), anti-TNF PE/Cyanine7 (MP6-XT22)), BD Biosciences (anti-SLAMF6 BUV395 (13G3), anti-KLRG1 Brilliant violet 650 (2F1), anti-CD103 Brilliant violet 786 (2E7)), Cell Signaling Technology (anti-TCF-1 Alexa Fluor 647 (C63D9)), and Ebioscience (anti-TOX PE (TXRX10)). Dead cells were excluded by staining with Zombie NIR dye (BioLegend). Most antibodies were used at a 1/100 dilution except for granzyme B (1/25). Peripheral blood samples were obtained by facial vein puncture in heparin. Blood erythrocytes were lysed, and cells fixed using BD FACS Lysing Solution (BD Biosciences, 349202). For the preparation of CNS infiltrating leukocytes, mice were anesthetized and transcardially perfused with PBS. Brains were minced, digested in DMEM with Collagenase A (1 mg/ml, Roche) and DNaseI (0.1 mg/ml, Roche) for 1 h at 37 °C and homogenized using 70-μm cell strainers (BD). Leukocytes were separated using a discontinuous percoll gradient (30/70%). The remaining erythrocytes were lysed using RBC Lysis buffer (BioLegend, 420301) for 3 min at RT. Surface staining was carried out with directly labeled antibodies in FACS buffer (2.5% FCS, 10 mM EDTA, 0.01% NaN3 in PBS). Isolated cells were quantified using AccuCheck Counting Beads (Invitrogen, PCB100). Intracellular staining of TCF-1 and TOX was performed using FoxP3/Transcription Factor Staining Buffer Set (eBioscience, 00-5523-00) according to manufacturer’s instructions. For ex vivo staining of Granzyme B, cells were fixed and permeabilized using commercial permabilization buffer set (BioLegend, 421002). To assess degranulation and intracellular cytokine production, brain leukocytes were cultured for 5 h in the presence of 5 μg/ml FITC labeled anti-CD107a antibody, monensin (BioLegend), and brefeldin (BioLegend). Cells were stimulated in vitro with 1 µM KAVYNFATC peptide. Cells were fixed and permeabilized using commercial fixation/permabilization buffer set (BioLegend, 421002) followed by intracellular staining for cytokines. Flow cytometric samples were acquired on a BD LSRFortessa (BD Biosciences) using BD FACSDiva (BD Biosciences, v8.0.2) and Attune NxT Acoustic Focusing Cytometer (Thermo Fisher, V3.1.2) using appropriate filter sets and compensation controls. Gates were assigned according to appropriate control populations (see Supplementary Fig. 7). In experiments which required high purity, P14 cells were sorted using Aria II flow cytometer within a laminar flow hood (BD Biosciences). Data were analyzed using FlowJo software (Treestar, V10).

### RNA-seq and ATAC-seq samples preparation

To perform RNA-seq, we FACS-sorted brain infiltrating P14 cells (*Tox*^+/+^ and *Tox*^−/−^) from WT and MOG-GP mice at different time points after rLCMV-GP33 infection. Each biological replicate (*n* = 3) represents 10^4^ sorted P14 cells sorted in RLT buffer with 1% ß-mercaptoethanol and processed with RNAeasy Micro Kit (Qiagen, 74004) following manufacturer’s instructions.

Quality and integrity of RNA were assessed with the fragment analyzer by using the standard sensitivity RNA Analysis Kit (Advanced Analytical, DNF-471). All samples selected for sequencing exhibited an RNA integrity number over 8. RNA-seq libraries were generated using 100 ng total RNA of a non-stranded RNA Seq, massively parallel mRNA sequencing approach from Illumina (TruSeq mRNA Library Preparation, Illumina, 20020594). Libraries preparation was performed on the Beckman Coulter’s Biomek FXP workstation. For accurate quantitation of cDNA libraries, the fluorometric based system QuantiFluor™dsDNA System was used (Promega, E2670). The size of final cDNA libraries was determined by using the dsDNA 905 Reagent Kit (Advanced Bioanalytical, DNF-905) exhibiting a sizing of 300 bp on average. Libraries were pooled and sequenced on the Illumina HiSeq 4000 sequencer (SE; 1 ×50 bp; 30–35 Mio reads/sample).

Basecalls generated by Illumina’s Real Time Analysis (RTA) software were demultiplexed to fastq files with bcl2fastq (v2.17.1.14). The quality check was done using FastQC (Andrews S. (2010). FastQC: a quality control tool for high throughput sequence data. Available online at http://www.bioinformatics.babraham.ac.uk/projects/fastqc) (version 0.11.8, Babraham Bioinformatics).

Each ATAC-seq was performed on two biological replicates of 5 × 10^4^ P14 cells (each replicate is a pool of P14 cells from ≥2 brains) as previously described^58^. Cells were spun down at 500 × *g* for 20 min at 4 °C. Nuclei were isolated in 50 μl of lysing solution composed of ﻿10 mM Tris-HCl, 10 mM NaCl, 3 mM MgCl_2_, and 0.1% IGEPAL CA-630. The nuclear pellet was resuspended into 50 μl of tagmentation buffer containing the Tn5 transposase (Illumina, 20034197) and incubated at 37 °C for 30 min. The tagmented DNA was purified using MiniElute PCR Purification Kit (Qiagen, 28004). Libraries were preamplified for 5 cycles using indexed primers (Supplementary Table 1) from Nextera kit (Illumina, FC-121-1031), and NEBNext High Fidelity PCR master mix (M0541S, New England Biolabs). Preamplified libraries served as input to determine the number of remaining cycles needed for the second round of PCR amplification. ﻿Libraries were cleaned up by AMPure XP Beads (Agencourt, A63880) at a 1.5× ratio.

Library molarity and quality were assessed with the Qubit (Thermofisher Scientific) and Tapestation using a DNA High sensitivity chip (Agilent Technologies, 5067-4626). Libraries were pooled at 2 nM and loaded for clustering on 1 lane of a Single-read Illumina Flow cell. Reads of 50 bases were generated using the TruSeq SBS chemistry on an Illumina HiSeq 4000 sequencer.

### RNA-seq data processing

FastQ reads were mapped to the ENSEMBL reference genome (GRCm38.89) using STAR version 2.4.0j^59^ with standard settings except that any reads mapping to more than one location in the genome (ambiguous reads) were discarded (*m* = 1).

A unique gene model was used to quantify the number of reads per gene. Briefly, the model considers all annotated exons of all annotated protein-coding isoforms of a gene to create a unique gene where the genomic regions of all exons are considered coming from the same RNA molecule and merged together.

All reads overlapping the exons of each unique gene model were reported using featureCounts version 1.4.6-p1^60^. Gene expressions were reported as raw counts and in parallel normalized in RPKM in order to filter out genes with low expression value (1 RPKM) before calling for differentially expressed genes. Library size normalizations and differential gene expression calculations were performed using the package edgeR (v3.28.0)^29^ designed for the R software (v3.6.2). Only genes having a significant fold change (Benjamini–Hochberg corrected *p*-value <0.05) were considered for the rest of the RNA-seq analysis.

### Pathway and GO term enrichment analysis

Gsea Preranked function of the gene set enrichment analysis (GSEA)^61^ was performed with gene sets identified using published microarray data sets. Effector and exhaustion signature originated from^24^ studying the transcriptome of distinct CD8+ T-cell differentiation states after LCMV Armstrong and LCMV clone 13 infection. To generate the effector gene list, we took the top 150 genes upregulated at day 8 post-LCMV Armstrong infection compared to naive. The exhaustion-related gene list were genes upregulated (Log_2_ FC ≥1; FDR < 0.05) in condition day 30 post-LCMV clone 13 infection compared to day 30 post-LCMV Armstrong infection. TCF-1-enriched gene signatures originated from microarray analysis of *Tcf7*^−/−^ and TCF-1-overexpressing CD8+ T cells^27^. GO terms (Biological Processes) and WikiPathways enrichment were performed with g:Profiler web tool^62^. The FDR threshold for statistical significance was set to 0.05.

### ATAC-seq data processing

ATAC-seq libraries were analyzed following the validated ENCODE pipeline from Kundaje lab (https://www.encodeproject.org/pipelines/ENCPL792NWO/). After adapter trimming with cutadapt version 1.9.1 (http://journal.embnet.org/index.php/embnetjournal/article/view/200/479), reads were mapped to the ENSEMBL reference genome (GRCm38.89) using bowtie2 version 2.2.6^63^. Duplicates were marked with MarkDuplicates function from picard-tools version 1.126 (http://broadinstitute.github.io/picard/) and removed using samtools view (-F 1804) version 1.2^64^. Library complexities were assessed using bedtools version 2.26.0 (https://bedtools.readthedocs.io/en/latest/) by calculating the ratios NRF (distinct/total), PBC1 (one read/distinct) and PBC2 (one read/two reads). Cross-correlations (NSC—Normalized Strand cross-correlation Coefficient and RSC—Relative Strand cross-correlation Coefficient) were calculated using the run_spp Rscript from Kundaje lab. For each biological replicate, two self-pseudoreplicates (containing half of the uniquely mapped reads) were generated following Kundaje lab recommendations. For each condition, pooled data of biological replicates and pooled-pseudoreplicates were created using zcat bash command. Reads on the forward and on the reverse strands were shifted by +4 bp and 5 bp, respectively, to account for the 9 bp duplication created by DNA repair of the nick by Tn5 transposase. Broad and narrow peaks were called using macs2 version v2.1.2 (https://github.com/taoliu/MACS) on each replicate, pseudoreplicate, pooled data, and pooled-pseudoreplicate, following Kundaje lab recommendations. Peaks overlapping problematic regions of the genome^65^ (http://mitra.stanford.edu/kundaje/akundaje/release/blacklists/mm10-mouse/mm10.blacklist.bed.gz) were removed using intersectBed from bedtools. Irreproducible discovery rate between each pair of biological replicates, pseudoreplicates, and pooled-pseudoreplicates was calculated using IDR 2.0.4 (https://github.com/nboley/idr). Optimal final peaks were selected using 5% threshold for IDR. Finally, FRiP (fraction of reads in peaks) were calculated using intersectBed from bedtools.

### Differential region accessibility

Differential region accessibility between two conditions was assessed by pooling all identified regions in both conditions. If two regions were overlapping by at least 50% of the width of the biggest region, these two regions were merged in a unique region corresponding to the overlap using the package GenomicRanges (v1.38.0) designed for R (http://web.mit.edu/~r/current/arch/i386_linux26/lib/R/library/GenomicRanges/html/findOverlaps-methods.html). Regions without overlap or overlapping by <50% were kept unchanged. A list of all identified regions within the two compared conditions was used to get read coverage in all biological replicates using featureCounts (v1.4.6)^66^. Coverages were then used as matrix input in EdgeR (v3.28.0)^29^ for normalization and differential region accessibility calculations. Distance between each pair of samples was assessed by the plotMDS function, representing the root-mean-square deviation (Euclidean distance) for the top 500 most variable regions. Localization of ChARs was performed with the function findOverlaps from the package GenomicRanges designed for R, using the previously described unique gene model. ChARs overlapping promoters (−2000 bp/+1000 bp of each unique gene model TSS) by at least 1 bp were considered localized in promoter regions. The rest of the peaks which overlapped exonic regions by at least 1 bp were assigned to exons. Then, the rest of the peaks overlapping intronic regions by at least 1 bp were assigned to introns. Finally, the rest of the peaks were assigned to intergenic regions. Same approach (overlap by at least 1 bp) was used to assign ChARs to exhaustion and effector-related peaks from ref. ^33^.

After normalization by total number of uniquely mapped reads in each replicate, coverages were represented by the z-score values for each exhaustion and effector-related peak.

### Heatmaps ATAC-seq and clustering

ChARs with abs(FC) ≥ 2 and *p*-value <0.05 (Fisher-exact test followed by Benjamini–Hochberg FDR correction) in the comparisons of interest were selected and extended by 500 bp upstream and downstream of their center. Read coverage at each bp in selected ChARs in each biological replicate was assessed using coverageBed from bedtools. For each replicate, ChAR coverages were normalized by the number of uniquely mapped reads. For each ChAR, signals among all replicates were scaled from 0 to 1. After hierarchical clustering of all selected ChARs using the function hclust() in R, the number of clusters was assessed by cutting the dendrogram in 2 to 20 clusters using the function cutree in R. The number of clusters (*k* = 5) was selected visually as the optimal number of clusters being the lowest number reporting correctly the variability of ChARs.

### Transcription factor binding site enrichment

Previously clustered ChAR sequences were extracted using the function subseq from the package Biostrings (https://bioconductor.org/packages/release/bioc/html/Biostrings.html) in R. All identified ChARs with abs(FC) < 1 and *p*-value >0.05 (Fisher-exact test followed by Benjamini–Hochberg FDR correction) and distant by at least 2000 bp to any clustered ChARs were used as background sequences taking as well 500 bp upstream and downstream of their centers (93,190 sequences). Transcription factor binding site (TFBS) enrichment vs. background sequences was tested for each cluster using findMotifs.pl script from HOMER (v4.9.1) (http://homer.ucsd.edu/homer/motif/). Only TFBS with a *p*-value below 10^−3^ (hypergeometric test) were represented.

### Assignment of ATAC-seq peaks to gene

Genes overlapping ChARs were automatically assigned to it. ChARs without overlap with any annotated genes on GRCm38.89 were assigned to the closest gene with a limit of 50 kb using the function nearest from the GenomicRanges package from R. TCF-1 binding site list was extracted from ref. ^41^. Correspondences on GRCm38.89 (mm10) were obtained with the hgLiftOver tool from UCSC (https://genome.ucsc.edu/cgi-bin/hgLiftOver). Genes having a TCF-1 identified binding site within 10 kb (upstream and/or downstream) were considered as TCF-1 bound genes. RNA-seq expression levels per gene and condition were extracted and scaled from 0 to 1 for each gene. Normalized levels of expression for genes having values in all conditions were reported on violin plots using the package vioplot (https://cran.r-project.org/web/packages/vioplot/index.html) designed for R. Genes concerned by several ChARs in one cluster were plotted only once.

### Combining genes with ATAC-seq regions

To evaluate the functional relevance of epigenetic changes mediated by TOX, a diamond plot was generated. In this plot, top 50 up- and downregulated genes which possess at least one differential ChAR (*p* < 0.05; Fisher-exact test followed by Benjamini–Hochberg FDR correction) within a 15 kb distance were selected and represented by a pile of diamonds. Each diamond represents a ChAR assigned to a gene. Color of each diamond represents the previously calculated gain or loss of accessibility (Log_2_ FC) of each ChAR in the comparison *Tox*^+/+^ vs. *Tox*^−/−^ A_L_ cells.

### Statistical analysis

Statistical parameters including the exact value of *n*, the dispersion, the precision of measures, and the statistical significance are reported in the figures and figure legends. In figures, asterisks denote statistical significance as calculated by Student’s *t* test and one-way ANOVA with Tukey’s post-test (ns, not significant; *p < 0.05; **p < 0.01; ***p < 0.001). Statistical analysis was performed in GraphPad Prism 8.

### Reporting summary

Further information on research design is available in the Nature Research Reporting Summary linked to this article.

## Supplementary information

Supplementary Information

Description of Additional Supplementary Files

Supplementary Data 1

Supplementary Data 2

Supplementary Data 3

Supplementary Data 4

Supplementary Data 5

Supplementary Data 6

Supplementary Data 7

Supplementary Data 8

Supplementary Data 9

Supplementary Data 10

Reporting Summary

## Data Availability

RNA-seq and ATAC-seq data have been deposited in the Gene Expression Omnibus (GEO) under the primary accession code: GSE149643. All other data are available in the article and its Supplementary Information files or from the corresponding author upon reasonable request. Source data are provided with this paper.

## Source data

Source Data

## Code availability

The ATAC-seq pipeline code is part of the ENCODE Uniform Processing Pipelines series and freely available on Github.
