# Supplementary material for: Persistence of self-reactive CD8+ T cells in the CNS requires TOX-dependent chromatin remodeling
Source: Nat Commun. 2021 Feb 12;12:1009. doi: 10.1038/s41467-021-21109-3 (PMC7881115; doi:10.1038/s41467-021-21109-3)
Supplement: Supplementary file 1 — Supplementary Information [file 41467_2021_21109_MOESM1_ESM.pdf]

Supplementary information

Persistence of self-reactive CD8+ T cells in the CNS requires TOX-dependent chromatin remodeling

Authors

Nicolas Page[1], Sylvain Lemeille[1], Ilena Vincenti[1], Bogna Klimek[1], Alexandre Mariotte[1], Ingrid Wagner[1], Giovanni Di Liberto[1], Jonathan Kaye[2], Doron Merkler[1,3,*]

Affiliations

[1] Department of Pathology and Immunology, University of Geneva, Geneva, Switzerland

[2] Research Division of Immunology, Departments of Biomedical Sciences and Medicine, Samuel Oschin Comprehensive Cancer Institute, Cedars-Sinai Medical Center, Los Angeles, CA, USA

[3]Division of Clinical Pathology, Geneva University Hospital, Geneva, Switzerland

[*]Corresponding author

Email: doron.merkler@unige.ch

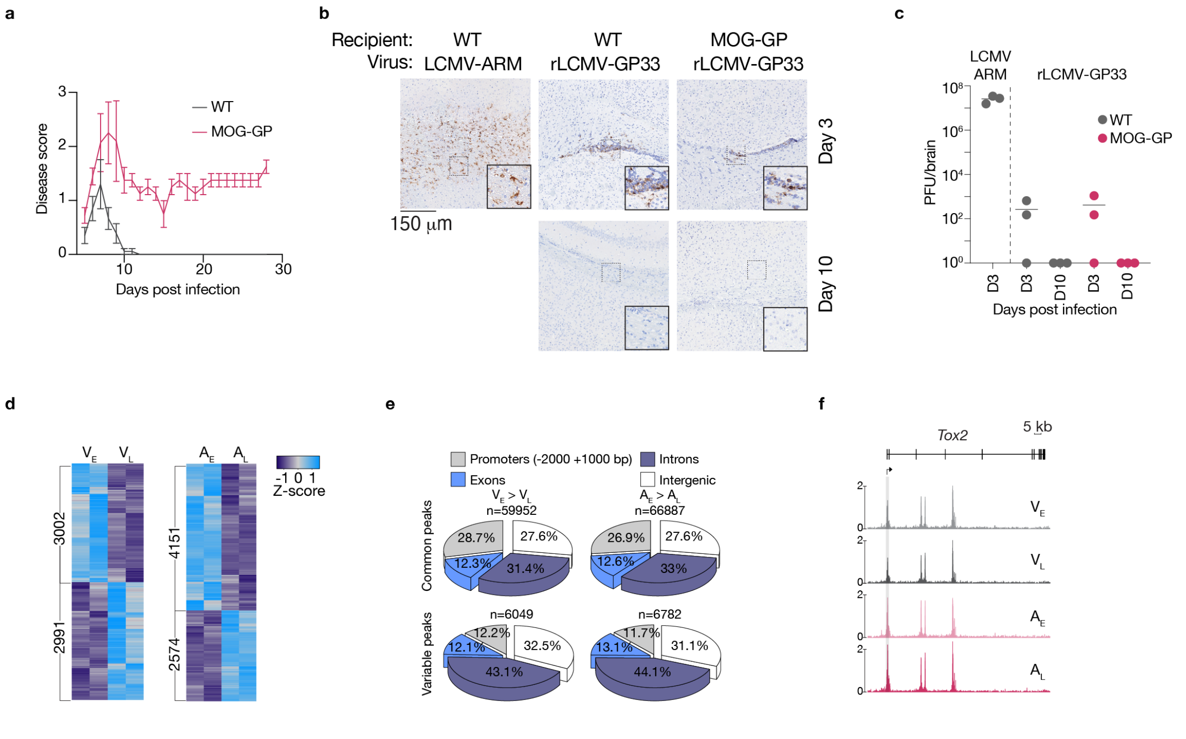

Supplementary Fig. 1 Chromatin accessibility changes in self-reactive CD8+ T cells

10^4 naïve P14 cells were adoptively transferred into WT and MOG-GP mice. One day later (day 0), mice were challenged i.c. with 10^4 PFU rLCMV-GP33. Brain infiltrating P14 cells were submitted to ATAC-seq 7 (V_E and A_E) and 21 (V_L and A_L) days after i.c. infection.

(**a**) EAE disease course (n = 10 WT mice and n= 9 MOG-GP mice), clinical scores are expressed as mean ± SEM.

(**b**) Brain sections of indicated experimental groups stained for LCMV-NP antigen.

(**c**) Viral titer determination in the brain of indicated experimental groups (n = 3 mice/group). Horizontal lines indicate the mean.

(**d**) Heatmap of the ATAC-seq Z-score of significantly differentially accessible ChARs in the comparisons (V_E versus V_L) and (A_E versus A_L). Variable peaks: (Log_2 FC $\geq$ 1; FDR < 0.05).

(**e**) Pie charts showing the distribution for common and variably accessible peaks within promoters, exons, introns and intergenic regions in the comparisons (V_E versus V_L) and (A_E versus A_L). Variable peaks: (Log_2 FC $\geq$ 1; FDR $\leq$ 0.05).

(**f**) ATAC-seq track of *Tox2* locus for V_E, V_L, A_E and A_L. Differentially accessible ChARs (FDR < 0.05) are highlighted in grey. Source data are provided as a Source Data file.

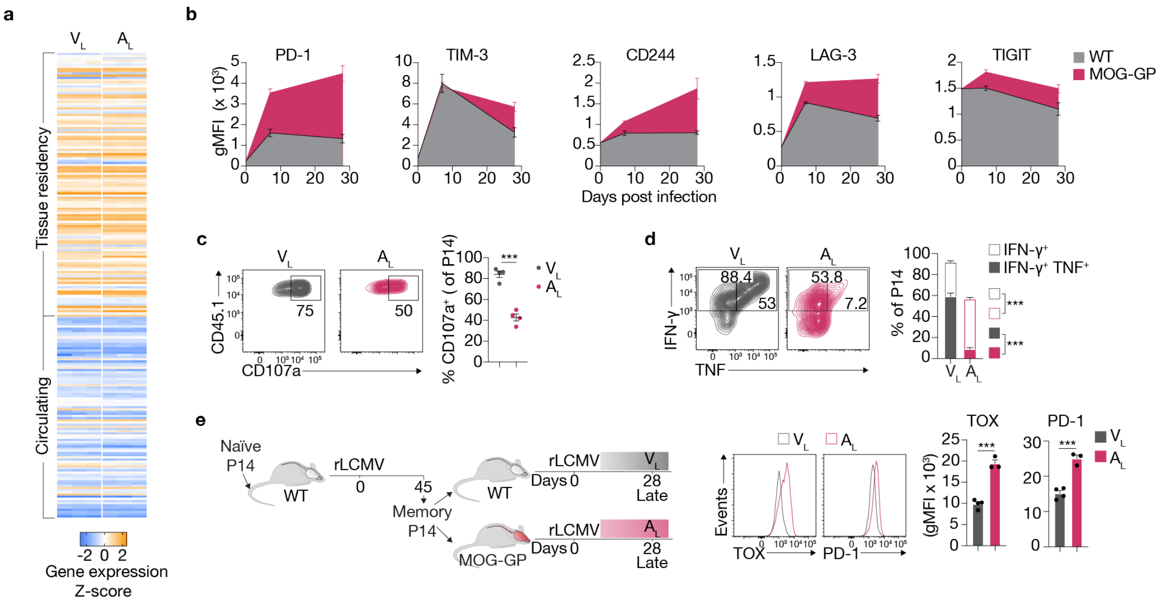

Supplementary Fig. 2 Self-reactive CD8+ T cells display a tissue-residency core signature and express exhaustion-associated markers

10^4 naïve P14 cells were adoptively transferred into WT and MOG-GP mice. One day later (day 0), mice were challenged i.c. with 10^4 PFU rLCMV-GP33. Brain infiltrating P14 cells were isolated for RNA-seq at day 21 after i.c. infection (a) and flow cytometric analysis at indicated days post infection (b-d).

(**a**) Heatmap showing the relative expression of transcripts belonging to core residency and core circulating program [1] in V_L and A_L P14 cells.

(**b**) Kinetic of inhibitory receptor expression in brain infiltrating P14 cells isolated from WT and MOG-GP mice (n = 3 mice/group at day 7 and n = 4 mice/group at day 28).

(**c**) Frequency of CD107a+ degranulating V_L and A_L cells after *in vitro* stimulation with KAVYNFATC peptide. Representative flow cytometry histograms (left) and summary data (right) (n = 4 mice/group; p = 0.0001).

(**d**) Intracellular staining for IFN-γ and TNF V_L (n = 3 mice) and A_L (n = 4 mice) cells at day 28 post-infection after *in vitro* stimulation with KAVYNFATC peptide. Numbers indicate the frequency of cytokine producing cells within each quadrant. Representative flow cytometry plots (left) and summary data (right). IFN-γ^+ (p = 0.0002); IFN-γ^+ + TNF (p < 0.0001).

(**e**) C57BL/6 hosts received P14 cells and were infected intravenously (i.v.) with rLCMV-GP33. 10^4 memory P14 cells isolated from the spleen 45 days after infection were transferred into WT (n = 4) and MOG-GP (n= 3) mice. One day later (day 0), mice were challenged i.c. with 10^4 PFU rLCMV-GP33. Brain infiltrating P14 cells were isolated for flow cytometric analysis of TOX (p = 0.0001) and PD-1 (p = 0.0005) expression at 28 days post infection in V_L and A_L. Experimental scheme (left) and FACS histograms and summary data (right). gMFI: geometric mean fluorescence intensity.

***p ≤ 0.001 (two-tailed unpaired t test for c-e). Data are representative of at least 2 independent experiments (b-e). Bars and horizontal lines represent mean ± SEM. Source data are provided as a Source Data file.

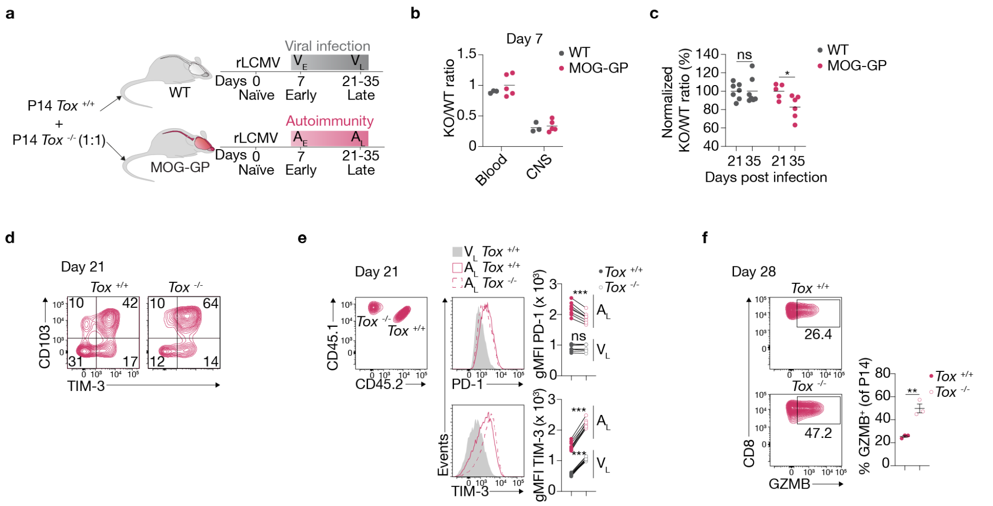

Supplementary Fig. 3 Cell intrinsic effect of TOX on self-reactive CD8+ T cell persistence and exhaustion phenotype

10^4 naïve *Tox* $^{+/+}$ and *Tox* $^{-/-}$ P14 cells were adoptively transferred at 1:1 ratio into WT and MOG-GP mice. One day later (day 0), mice were challenged i.c. with 10^4 PFU rLCMV-GP33. P14 cells were analyzed at day 7 (b), day 21/35 (c), and day 21 (e) after infection for flow cytometric analysis.

(**a**) Experimental scheme.

(**b**) Ratios of *Tox* $^{-/-}$/*Tox* $^{+/+}$ P14 cells in the blood and the brain 7 days after rLCMV-GP33 infection (n = 3 WT mice and n = 5 MOG-GP mice).

(**c**) Normalized ratios of *Tox* $^{-/-}$/*Tox* $^{+/+}$ P14 cells recovered from the brain between day 21(n = 7 WT mice; n = 5 MOG-GP mice) to 35 (n = 7 WT mice; n = 6 MOG-GP mice) after infection. The ratios of *Tox* $^{-/-}$/*Tox* $^{+/+}$ P14 cells recovered at day 21 after infection were set up at 100% for each group. WT (p = 0.9745); MOG-GP (p = 0.0285).

(**d**) 10^4 naïve *Tox* $^{+/+}$ or *Tox* $^{-/-}$ P14 cells were adoptively transferred into MOG-GP mice. One day later (day 0), mice were challenged i.c. with 10^4 PFU rLCMV-GP33. Brain infiltrating P14 cells were isolated at 21 days post infection for flow cytometric analysis of CD103 and TIM-3 expression.

(**e**) Expression of PD-1 and TIM-3 in *Tox* $^{+/+}$ and *Tox* $^{-/-}$ A_L and V_L P14 cells. Gating strategy and representative flow cytometry histograms (left) and summary data (right) (n = 8 mice/group). PD-1 (A_L, $p < 0.0001$; V_L, $p = 0.2672$); TIM-3 (A_L, $p < 0.0001$; V_L, $p < 0.0001$).

(**f**) 10^4 naïve *Tox* $^{+/+}$ or *Tox* $^{-/-}$ P14 cells were adoptively transferred into MOG-GP mice. One day later (day 0), mice were challenged i.c. with 10^4 PFU rLCMV-GP33. Brain infiltrating P14 cells were isolated at 28 days post infection for flow cytometric analysis of granzyme B expression. Representative flow cytometry plots (left) and summary data (right) (n = 3 mice/group; p = 0.0035). Horizontal lines represent mean ± SEM.

ns, not significant; *p ≤ 0.05; **p ≤ 0.01; ***p ≤ 0.001 (two-tailed unpaired t test for c, f and two-tailed paired t test for e). Data are representative of at least 2 independent experiments (e-f). Source data are provided as a Source Data file.

a

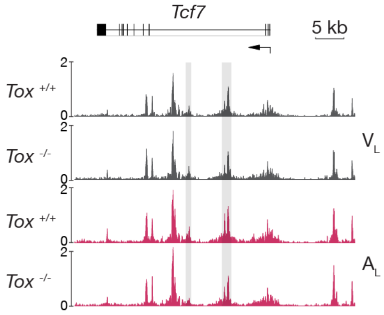

Supplementary Fig. 4 TOX epigenetically remodels *Tcf7* locus in self-reactive CD8+ T cells

10^4 naïve *Tox* $^{+/+}$ or *Tox* $^{-/-}$ P14 cells were adoptively transferred into WT and MOG-GP mice. One day later (day 0), mice were challenged i.c. with 10^4 PFU rLCMV-GP33. Brain infiltrating P14 cells were FACS sorted and submitted to ATAC-seq 21 days later.

(**a**) ATAC-seq track of *Tcf7* locus for *Tox* $^{+/+}$ and *Tox* $^{-/-}$ V_L and A_L P14 cells. Differentially accessible ChARs (FDR $\leq$ 0.05) are highlighted in grey.

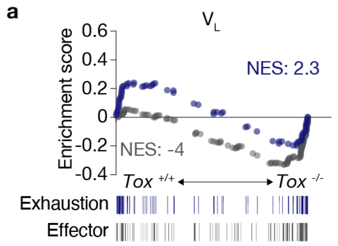

Supplementary Fig. 5 TOX controls the expression of effector associated genes in CD8+ T cells

10^4 naïve *Tox* $^{+/+}$ or *Tox* $^{-/-}$ P14 cells were adoptively transferred into WT mice. One day later (day 0), mice were challenged i.c. with 10^4 PFU rLCMV-GP33. Brain infiltrating P14 cells were FACS sorted for RNA-seq 21 days later.

(**a**) GSEA of a signature of exhaustion and effector differentiation [2] in a ranked list of genes differentially expressed by *Tox* $^{+/+}$ versus *Tox* $^{-/-}$ V_L cells. NES: normalized enrichment score.

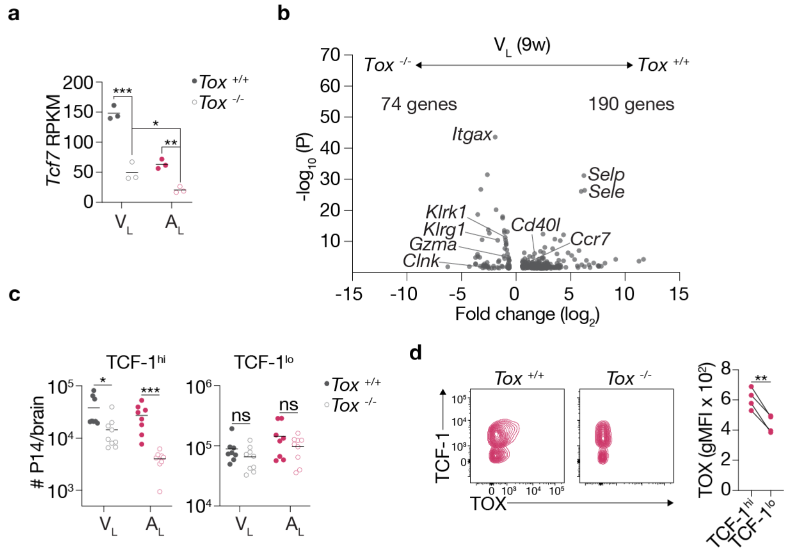

Supplementary Fig. 6 TOX predominantly preserves the pool of self-reactive TCF-1^{hi} CD8+ T cells

10^4 naïve *Tox* $^{+/+}$ or *Tox* $^{-/-}$ P14 cells were adoptively transferred into WT and MOG-GP mice. One day later (day 0), mice were challenged i.c. with 10^4 PFU rLCMV-GP33 and brain infiltrating P14 cells were isolated for RNA-seq (a-b) or FACS analysis (c-d) 3 weeks after infection.

(**a**) RPKM values of *Tcf7* in *Tox* $^{+/+}$ versus *Tox* $^{-/-}$ V_L and A_L (n = 3 mice/group). Horizontal lines represent the mean. *Tox* $^{+/+}$ versus *Tox* $^{-/-}$ V_L ($p < 0.0001$); *Tox* $^{+/+}$ versus *Tox* $^{-/-}$ A_L ($p = 0.0051$); *Tox* $^{-/-}$ V_L versus *Tox* $^{-/-}$ A_L ($p = 0.0415$).

(**b**)10^4 naïve *Tox* $^{+/+}$ or *Tox* $^{-/-}$ P14 cells were adoptively transferred into WT mice. One day later (day 0), mice were challenged i.c. with 10^4 PFU rLCMV-GP33 and brain infiltrating P14 cells were isolated for RNA-seq analysis at 9 weeks after infection.

Volcano plot of differentially expressed genes (DEGs) (FC $\geq$ 1.5; FDR < 0.05) in *Tox* $^{+/+}$ versus *Tox* $^{-/-}$ V_L cells at 9 weeks after infection (n = 3 mice/group).

(**c**) Enumeration of TCF-1^{hi} and TCF-1^{lo} in V_L (*Tox* $^{+/+}$, n = 8; *Tox* $^{-/-}$, n = 9) and A_L (*Tox* $^{+/+}$, n = 8; *Tox* $^{-/-}$, n = 9) cells. TCF-1^{hi} (V_L, p = 0.0206; A_L, p = 0.0003); TCF-1^{lo} (V_L, p = 0.2313; A_L, p = 0.1933). Horizontal lines represent the mean.

(**d**) TOX expression in TCF-1^{hi} and TCF-1^{lo} subsets of A_L cells. Representative flow cytometry plots (left; *Tox* $^{-/-}$ P14 cells were included as control staining) and summary data (right) (n = 4 mice/group; p = 0.0026).

ns, not significant; *$p \leq 0.05$; **$p \leq 0.01$; ***$p \leq 0.001$ (one-way ANOVA with Tukey's post-test for a and two-tailed unpaired t test for c and two-tailed paired t test for d).

Data represent the pool of 2 independent experiments (c) or are representative of at least 2 independent experiments (d). Source data are provided as a Source Data file.

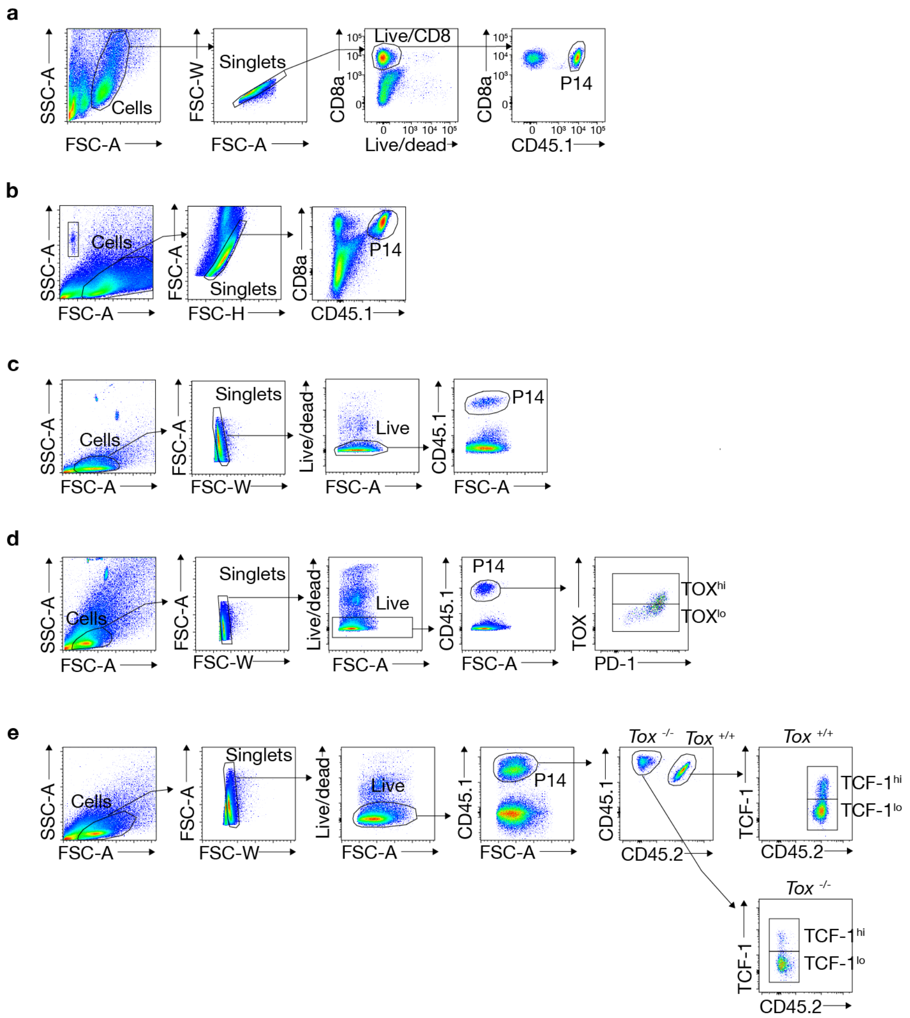

Supplementary Fig. 7 Gating strategies used for cell sorting and phenotyping of P14 cells

(**a**) Gating strategy used to sort brain infiltrating P14 cells for ATAC-seq and RNA-seq analysis presented in Fig. 1, 2a-d, 4, 5, 6a, 6c and Supp. Fig. 1d-f, 2a, 4, 5, 6a-b.

(**b**) Gating strategy used to investigate the phenotype (Fig. 2e, 3e, Supp. Fig. 2b-d, 3f, 6d) and the number (Fig. 3a) of brain infiltrating P14 cells in WT and MOG-GP mice.

(**c**) Gating strategy used to determine the percentage and absolute counts of TCF-1^{hi} versus TCF-1^{lo} P14 cells (Fig. 6b and Supp. Fig. 6c) or protein expression in brain infiltrating P14 cells (Fig. 2f, 3b-d, Supp. Fig. 2e, 3d).

(**d**) Gating strategy used to stratify marker expression on TOX^{hi} and TOX^{lo} P14 cells as presented in Fig. 2g.

(**e**) Gating strategy used to phenotype congenically distinct P14 cells in co-transfer experiment (Fig. 2e). The same strategy was used to additionally stratify co-transferred P14 cells based on TCF-1 expression (Fig. 6d).

Ad1_noMX	AATGATACGGCGACCACCGAGATCTACACTCGTCGGCAGCGTCAGATGTG
Ad2.1_TAAGGCGA	CAAGCAGAAGACGGCATACGAGATTCGCCTTAGTCTCGTGGGCTCGGAGATGT
Ad2.2_CGTACTAG	CAAGCAGAAGACGGCATACGAGATCTAGTACGGTCTCGTGGGCTCGGAGATGT
Ad2.3_AGGCAGAA	CAAGCAGAAGACGGCATACGAGATTTCTGCCTGTCTCGTGGGCTCGGAGATGT
Ad2.4_TCCTGAGC	CAAGCAGAAGACGGCATACGAGATGCTCAGGAGTCTCGTGGGCTCGGAGATGT
Ad2.5_GGACTCCT	CAAGCAGAAGACGGCATACGAGATAGGAGTCCGTCTCGTGGGCTCGGAGATGT
Ad2.6_TAGGCATG	CAAGCAGAAGACGGCATACGAGATCATGCCTAGTCTCGTGGGCTCGGAGATGT
Ad2.7_CTCTCTAC	CAAGCAGAAGACGGCATACGAGATGTAGAGAGGTCTCGTGGGCTCGGAGATGT
Ad2.8_CAGAGAGG	CAAGCAGAAGACGGCATACGAGATCCTCTCTGGTCTCGTGGGCTCGGAGATGT

Supplementary Table 1 Nextera indexed primers used for ATAC-seq library amplification

Supplementary References

1. Milner, J.J. et al. Runx3 programs CD8(+) T cell residency in non-lymphoid tissues and tumours. Nature 552, 253-257 (2017).

2. Doering, T.A. et al. Network analysis reveals centrally connected genes and pathways involved in CD8+ T cell exhaustion versus memory. Immunity 37, 1130-1144 (2012).
