# Supplementary material for: Persistence of self-reactive CD8+ T cells in the CNS requires TOX-dependent chromatin remodeling
Source: Nat Commun. 2021 Feb 12;12:1009. doi: 10.1038/s41467-021-21109-3 (PMC7881115; doi:10.1038/s41467-021-21109-3)
Supplement: Supplementary file 2 — Description of Additional Supplementary Files [file 41467_2021_21109_MOESM2_ESM.pdf]

Description of Additional Supplementary Files

Supplementary Data 1. Table depicting differential chromatin accessibility in the following pairwise comparisons: VL versus VE, AL versus AE, AE versus VE, and AL versus VL. Significance were determined by Fisher-exact test followed by Benjamini-Hochberg FDR correction.

Supplementary Data 2. Table depicting the clustering of differentially accessible ChARs obtained in the comparison VE versus AE and/or VL versus AL (Figure 1e). Relative peak intensity of each ChAR is indicated.

Supplementary Data 3. Transcription factor binding motif enrichment using HOMER in clusters identified in Figure 1e and Supplementary Data 2.

Supplementary Data 4. Differential gene expression analysis in the comparison VL versus AL depicted in Figure 2a. Significance were determined by Fisher-exact test followed by Benjamini-Hochberg FDR correction.

Supplementary Data 5. Table depicting the Log2 fold change of differentially expressed genes (FDR < 0.05) found adjacent to differentially accessible ChARS (Log2 FC ^{3} 1; FDR < 0.05). ATAC-seq Z-scores of ChARs and their respective distance to an adjacent gene are indicated.

Supplementary Data 6. Table depicting TOX-dependent chromatin accessibility changes in the following pairwise comparisons: Tox -/- versus Tox +/+ VL and Tox -/- versus Tox +/+ AL. Significance were determined by Fisher-exact test followed by Benjamini-Hochberg FDR correction.

Supplementary Data 7. Table depicting the clustering of TOX-dependent differentially accessible ChARs in VE and/or AE based on their relative peak intensity.

Supplementary Data 8. Transcription factor binding motif enrichment using HOMER in clusters identified in Figure 4e and Supplementary Data 7.

Supplementary Data 9. Differential gene expression analysis in the comparison Tox -/- versus Tox +/+ AL (3 weeks post-infection), Tox -/- versus Tox +/+ VL (3 weeks post-infection), and Tox -/- versus Tox +/+ VL (9 weeks post-infection). Significance were determined by Fisher-exact test followed by Benjamini-Hochberg FDR correction.

Supplementary Data 10. Gene expression changes associated with TCF-1 binding in ATAC-seq clusters identified in Figure 4d and Supplementary Data 7.
