# Supplementary material for: Persistence of self-reactive CD8+ T cells in the CNS requires TOX-dependent chromatin remodeling
Source: Nat Commun. 2021 Feb 12;12:1009. doi: 10.1038/s41467-021-21109-3 (PMC7881115; doi:10.1038/s41467-021-21109-3)
Supplement: Supplementary file 13 — Reporting Summary [file 41467_2021_21109_MOESM13_ESM.pdf]

nature research

Corresponding author(s): Pr. Doron Merkler

Last updated by author(s): Dec 16, 2020

Reporting Summary

Nature Research wishes to improve the reproducibility of the work that we publish. This form provides structure for consistency and transparency in reporting. For further information on Nature Research policies, see our Editorial Policies and the Editorial Policy Checklist.

Statistics

For all statistical analyses, confirm that the following items are present in the figure legend, table legend, main text, or Methods section.

n/a	Confirmed	
☐	☒	The exact sample size (*n*) for each experimental group/condition, given as a discrete number and unit of measurement
☐	☒	A statement on whether measurements were taken from distinct samples or whether the same sample was measured repeatedly
☐	☒	The statistical test(s) used AND whether they are one- or two-sided<br>*Only common tests should be described solely by name; describe more complex techniques in the Methods section.*
☒	☐	A description of all covariates tested
☐	☒	A description of any assumptions or corrections, such as tests of normality and adjustment for multiple comparisons
☐	☒	A full description of the statistical parameters including central tendency (e.g. means) or other basic estimates (e.g. regression coefficient) AND variation (e.g. standard deviation) or associated estimates of uncertainty (e.g. confidence intervals)
☐	☒	For null hypothesis testing, the test statistic (e.g. *F*, *t*, *r*) with confidence intervals, effect sizes, degrees of freedom and *P* value noted<br>*Give P values as exact values whenever suitable.*
☒	☐	For Bayesian analysis, information on the choice of priors and Markov chain Monte Carlo settings
☒	☐	For hierarchical and complex designs, identification of the appropriate level for tests and full reporting of outcomes
☒	☐	Estimates of effect sizes (e.g. Cohen's *d*, Pearson's *r*), indicating how they were calculated

Our web collection on statistics for biologists contains articles on many of the points above.

Software and code

Policy information about availability of computer code

Data collection	Flow cytometry data were collected using BD FACSDiva (v8.0.2) and Attune NxT Acoustic Focusing Cytometer software (Thermo Fisher, V3.1.2).<br>RNA-seq libraries were pooled and sequenced on the Illumina HiSeq 4000 sequencer (SE; 1 x 50 bp; 30-35 Mio reads/sample).<br>Basecalls generated by Illumina's Real Time Analysis (RTA) software were demultiplexed to fastq files with bcl2fastq v2.17.1.14. The quality check was done using FastQC (version 0.11.8, Babraham Bioinformatics).<br>ATAC-seq reads (SE; 1 x 50 bp) were generated using the TruSeq SBS chemistry on an Illumina HiSeq 4000 sequencer.
Data analysis	Flow cytometry data were analyzed on FlowJo v10 (TreeStar) and statistical analysis performed on Prism v8 (GraphPad).<br>For RNA-seq processing, FastQ reads were mapped to the ENSEMBL reference genome (GRCm38.89) using STAR version 2.4.0j with standard settings except that any reads mapping to more than one location in the genome (ambiguous reads) were discarded (m=1).<br>A unique gene model was used to quantify the number of reads per gene. Briefly, the model considers all annotated exons of all annotated protein coding isoforms of a gene to create a unique gene where the genomic regions of all exons are considered coming from the same RNA molecule and merged together.<br>All reads overlapping the exons of each unique gene model were reported using featureCounts version 1.4.6-p1 54. Gene expressions were reported as raw counts and in parallel normalized in RPKM in order to filter out genes with low expression value (1 RPKM) before calling for differentially expressed genes. Library size normalizations and differential gene expression calculations were performed using the package edgeR (v3.28.0) designed for the R software.<br>ATAC-seq libraries were analyzed following the validated ENCODE pipeline from Kundaje lab (https://www.encodeproject.org/pipelines/ENCPL792NWO/). After adapter trimming with cutadapt version 1.9.1 (http://journal.embnet.org/index.php/embnetjournal/article/view/200/479), reads were mapped to the ENSEMBL reference genome (GRCm38.89) using bowtie2 version 2.2.6 58. Duplicates were marked with MarkDuplicates function from picard-tools version 1.126 (http://broadinstitute.github.io/picard/) and removed using samtools view (-F

1804) version 1.2 59. Library complexities were assessed using bedtools version 2.26.0 (https://bedtools.readthedocs.io/en/latest/) by calculating the ratios NRF (distinct/total), PBC1 (one read/distinct) and PBC2 (one read/two reads). Cross-correlations (NSC - Normalized Strand cross-correlation Coefficient and RSC – Relative Strand cross-correlation Coefficient) were calculated using the run_spp Rscript from Kundaje lab. For each biological replicate, two self-pseudoreplicates (containing half of uniquely mapped reads) were generated following Kundaje lab recommendations. For each condition, pooled data of biological replicates and pooled-pseudoreplicates were created using zcat bash command. Reads on the forward and on the reverse strands were shifted by +4bp and 5bp respectively to account for the 9bp duplication created by DNA repair of the nick by Tn5 transposase. Broad and narrow peaks were called using macs2 version v2.1.2 (https://github.com/taoliu/MACS) on each replicate, pseudoreplicate, pooled data and pooled-pseudoreplicate, following Kundaje lab recommendations. Peaks overlapping problematic regions of the genome (http://mitra.stanford.edu/kundaje/akundaje/release/blacklists/mm10-mouse/mm10.blacklist.bed.gz) were removed using intersectBed from bedtools. Irreproducible Discovery Rate between each pair of biological replicates, pseudoreplicates and pooled-pseudoreplicates was calculated using IDR 2.0.4 (https://github.com/nboley/idr). Optimal final peaks were selected using 5% threshold for IDR. Finally, FRiP (Fraction of Reads in Peaks) were calculated using intersectBed from bedtools.

Differential region accessibility between two conditions was assessed by pooling all identified regions in both conditions. If two regions were overlapping by at least 50% of the width of the biggest region, these two regions were merged in a unique region corresponding to the overlap using the package GenomicRanges (v1.38.0) designed for R (v3.6.2) (http://web.mit.edu/~r/current/arch/i386_linux26/lib/R/library/GenomicRanges/html/findOverlaps-methods.html). Regions without overlap or overlapping by less than 50% were kept unchanged. A list of all identified regions within the two compared conditions was used to get read coverage in all biological replicates using featureCounts version 1.4.6 68. Coverages were then used as matrix input in EdgeR (v3.28.0) for normalization and differential region accessibility calculations. Distance between each pair of samples was assessed by the plotMDS function, representing the root-mean-square deviation (Euclidean distance) for the top 500 most variable regions. Localization of ChARs was performed with the function findOverlaps from the package GenomicRanges designed for R, using the previously described unique gene model. ChARs overlapping promoters (-2000bp / + 1000bp of each unique gene model TSS) by at least 1bp were considered localized in promoter regions. The rest of the peaks which overlapped exonic regions by at least 1bp were assigned to exons. Then, the rest of the peaks overlapping intronic regions by at least 1bp were assigned to introns. Finally, the rest of the peaks were assigned to intergenic regions. Same approach (overlap by at least 1bp) was used to assign ChARs to exhaustion and effector-related peaks.

After normalization by total number of uniquely mapped reads in each replicate, coverages were represented by the z-score values for each exhaustion and effector-related peak.

ChARs with abs(FC) ≥ 2 and pvalue < 0.05 in the comparisons of interest were selected and extended by 500bp upstream and downstream of their center. Read coverage at each bp in selected ChARs in each biological replicate was assessed using coverageBed from bedtools. For each replicate, ChAR coverages were normalized by the number of uniquely mapped reads. For each ChAR, signals among all replicates were scaled from 0 to 1. After hierarchical clustering of all selected ChARs using the function hclust() in R, the number of clusters was assessed by cutting the dendrogram in 2 to 20 clusters using the function cutree in R. The number of clusters (k=5) was selected visually as the optimal number of clusters being the lowest number reporting correctly the variability of ChARs. Previously clustered ChAR sequences were extracted using the function subseq from the package Biostrings (https://bioconductor.org/packages/release/bioc/html/Biostrings.html) in R. All identified ChARs with abs(FC) < 1 and pvalue > 0.05 and distant by at least 2000bp to any clustered ChARs were used as background sequences taking as well 500bp upstream and downstream of their centers (93’190 sequences). Transcription factor binding site (TFBS) enrichment versus background sequences was tested for each cluster using findMotifs.pl script from HOMER (v4.9.1) (http://homer.ucsd.edu/homer/motif/).

Genes overlapping ChARs were automatically assigned to it. ChARs without overlap with any annotated genes on GRCm38.89 were assigned to the closest gene with a limit of 50kb using the function nearest from the GenomicRanges package from R. TCF-1 binding site list was extracted from reference 42. Correspondences on GRCm38.89 (mm10) were obtained with the hgLiftOver tool from ucsc (https://genome.ucsc.edu/cgi-bin/hgLiftOver). Genes having a TCF-1 identified binding site within 10kb (upstream and/or downstream) were considered as TCF-1 bound genes. RNA-seq expression levels per gene and condition were extracted and scaled from 0 to 1 for each gene. Normalized levels of expression for genes having values in all conditions were reported on violin plots using the package vioplot (https://cran.r-project.org/web/packages/vioplot/index.html) designed for R. Genes concerned by several ChARs in one cluster were plotted only once.

For manuscripts utilizing custom algorithms or software that are central to the research but not yet described in published literature, software must be made available to editors and reviewers. We strongly encourage code deposition in a community repository (e.g. GitHub). See the Nature Research guidelines for submitting code & software for further information.

Data

Policy information about availability of data

All manuscripts must include a data availability statement. This statement should provide the following information, where applicable:

- Accession codes, unique identifiers, or web links for publicly available datasets
- A list of figures that have associated raw data
- A description of any restrictions on data availability

The data that support the findings of this study are available from the corresponding author upon reasonable request. The raw and processed RNA-seq and ATAC-seq data have been deposited to the Gene Expression Omnibus (GEO) under accession numbers GEO: GSE14643.

Field-specific reporting

Please select the one below that is the best fit for your research. If you are not sure, read the appropriate sections before making your selection.

☒ Life sciences ☐ Behavioural & social sciences ☐ Ecological, evolutionary & environmental sciences

For a reference copy of the document with all sections, see nature.com/documents/nr-reporting-summary-flat.pdf

Life sciences study design

All studies must disclose on these points even when the disclosure is negative.

Sample size	Animal sample size were determined using previous studies with intracranial LCMV infection model (Steinbach et al, 2016), the availability of the mice and based on 3R principle.
Data exclusions	No data were excluded from the analysis.
Replication	Experimental replication was performed as indicated in the figure legends and replications were successful.
Randomization	Mice were randomly distributed amongst groups at the start of each experiment.
Blinding	The investigators were not blinded during experimental procedure due to technical reasons. In particular, the procedures have to be clearly labeled on cage cards and scoring sheets as required by local authorities.

Reporting for specific materials, systems and methods

We require information from authors about some types of materials, experimental systems and methods used in many studies. Here, indicate whether each material, system or method listed is relevant to your study. If you are not sure if a list item applies to your research, read the appropriate section before selecting a response.

Materials & experimental systems

n/a	Involved in the study	
	x	Antibodies
	x	Eukaryotic cell lines
x		Palaeontology and archaeology
	x	Animals and other organisms
x		Human research participants
x		Clinical data
x		Dual use research of concern

Methods

n/a	Involved in the study	
x		ChIP-seq
	x	Flow cytometry
x		MRI-based neuroimaging

Antibodies

Antibodies used

Brillant violet 510 anti-mouse CD8a (clone 53-6.7) Biolegend Cat#100751; RRID: AB_2561389
Brillant violet 605 anti-mouse CD8a (clone 53-6.7) Biolegend Cat#100743, RRID: AB_2561352
PerCP/Cyanine5.5 anti-mouse CD244 (clone m2B4 (B6)458.1) Biolegend Cat#133513; RRID: AB_2564340
PE/Cyanine7 anti-mouse TIGIT (clone 1G9) Biolegend Cat#142107; RRID: AB_2565648
PE anti-mouse PD-1 (clone 29F.1A12) Biolegend Cat#135205; RRID: AB_1877232
PerCP/Cyanine5.5 anti-mouse PD-1 (clone 29F.1A12) Biolegend Cat#135207; RRID: AB_10550092
FITC anti-mouse PD-1 (clone 29F.1A12) Biolegend Cat#135213; RRID: AB_10689633
APC anti-mouse LAG-3 (clone C9B7W) Biolegend Cat#125209; RRID: AB_10639935
Alexa Fluor 700 anti-mouse CD45.1 (clone A20) Biolegend Cat#110723; RRID: AB_493732
Pacific Blue anti-mouse CD45.1 (clone A20) Biolegend Cat#110721 RRID: AB_492867
Brillant violet 785 anti-mouse CD45.1 (clone A20) Biolegend Cat#110743; RRID: AB_2563379
Brillant violet 605 anti-mouse TIM-3 (clone RMT3-23) Biolegend Cat#119721; RRID: AB_2616907
Brillant violet 711 anti-mouse TIM-3 (clone RMT3-23) Biolegend Cat#119727; RRID: AB_2716208
FITC anti-mouse CD107a (clone 1D4B) Biolegend Cat#121606; RRID: AB_572007
PE/Cyanine7 anti-mouse TNFa (clone MP6-XT22) Biolegend Cat#506323; RRID: AB_2204356
PE anti-mouse IL-2 (clone JES6-5H4) Biolegend Cat# 503807; RRID: AB_315301
APC anti-mouse IFNg (clone XMG1.2) Biolegend Cat#505809; RRID: AB_315403
Alexa Fluor 647 anti-mouse Granzyme B (clone GB11) Biolegend Cat#515405; RRID: AB_2294995
FITC anti-mouse KLRG1 (clone 2F1/KLRG1) Biolegend Cat#138409; RRID: AB_10643998
Brillant violet 421 anti-mouse KLRG1 (clone 2F1/KLRG1) Biolegend Cat#138413; RRID: AB_10918627
Brillant violet 650 anti-mouse KLRG1 (clone 2F1) BD Biosciences Cat#740553;RRID: AB_2740254
Alexa Fluor 647 anti-mouse TCF-1 (clone C63D9) Cell Signaling Technology Cat#6709; RRID: AB_2199302
PE anti-TOX (clone TXRX10) eBioscience Cat#12-6502-82; RRID: AB_10855034
PE/Cyanine7 anti-mouse CD45.2 (clone 104) Biolegend Cat# 109829; RRID: AB_1186103
BUV395 anti-mouse SLAMF6 (clone 13G3) BD Biosciences Cat# 745730; RRID: AB_2743205
Brillant violet 786 anti-mouse CD103 (clone 2E7) BD Biosciences Cat#748254; RRID: AB_2847906
Gossmann et al, 1991 - rabbit serum anti-LCMV nucleoprotein Immunohistochemistry
Generated in house - rat anti-LMCV (VL4) Focus forming assay

Validation	Please find below a table indicating the validation method of all antibodies used in this study.<br>Supplier Catalogue number Clone Validation method<br>Biolegend Cat#100751 53-6.7 C57BL/6 mouse splenocytess<br>Biolegend Cat#100743 53-6.7 C57BL/6 mouse splenocytess<br>Biolegend Cat#133513 m2B4(B6)458.1 C57BL/6 mouse splenocytess<br>Biolegend Cat#142107 1G9 Con-A stimulated mouse splenocytes<br>Biolegend Cat#135205 29F.1A12 Con-A stimulated mouse splenocytes<br>Biolegend Cat#135207 29F.1A12 Con-A stimulated mouse splenocytes<br>Biolegend Cat#135213 29F.1A12 Con-A stimulated mouse splenocytes<br>Biolegend Cat#125209 C9B7W Con-A stimulated mouse splenocytes<br>Biolegend Cat#110723 A20 SJL mouse splenocytes<br>Biolegend Cat#110721 A20 SJL mouse splenocytes<br>Biolegend Cat#110743 A20 SJL mouse splenocytes<br>Biolegend Cat#119721 RMT3-23 Mouse Tim-3 transfected cells<br>Biolegend Cat#119727 RMT3-23 Mouse Tim-3 transfected cells<br>Biolegend Cat#121606 1D4B Thioglycollate-elicited BALB/c mouse peritoneal macrophages<br>Biolegend Cat#506323 MP6-XT22 PMA + Ionomycin-stimulated C57BL/6 mouse splenocytes (in the presence of monensin)<br>Biolegend Cat#503807 JES6-5H4 PMA+ionomycin-stimulated C57BL/6 mouse splenocytes<br>Biolegend Cat#505809 XMG1.2 PMA/Ionomycin-stimulated (6hrs) C57BL/6 mouse splenocytes<br>Biolegend Cat#515405 GB11 Human peripheral blood lymphocytes<br>Biolegend Cat#138409 2F1 C57BL/6 mouse splenocytess<br>Biolegend Cat#138413 2F1 C57BL/6 mouse splenocytess<br>BD Biosciences Cat#740553 2F1 C57BL/6 mouse splenocytess<br>Cell Signaling Cat#6709 C63D9 Jurkat cells<br>eBioscience Cat#12-6502-82 TXRX10 C57Bl/6 thymocytes<br>Biolegend Cat#109829 104 C57BL/6 mouse splenocytess<br>BD Biosciences Cat#745730 13G3 C57BL/6 Mouse bone marrow cells and thymocytes<br>BD Biosciences Cat#748254 2E7 C57BL/6 mouse splenocytess<br>Gossmann et al, 1991 Rabbit serum Rabbit anti-LCMV Tissues from LCMV infected C57BL/6 mice and Naïve (non-infected)<br>Generated in house VL4 hybridoma VL4 Tissues from LCMV infected C57BL/6 mice and Naïve (non-infected)

Eukaryotic cell lines

Policy information about cell lines

Cell line source(s)	The MC57G (ATCC CRL-2295) and BHK21 (ATCC CCL-10) were purchased from the American Type Culture Collection.
Authentication	None of the cell lines use in this study were authenticated.
Mycoplasma contamination	All cell lines are mycoplasma-free.
Commonly misidentified lines (See ICLAC register)	No commonly misidentified cell lines were used in this study.

Animals and other organisms

Policy information about studies involving animals; ARRIVE guidelines recommended for reporting animal research

Laboratory animals	C57BL/6J WT were obtained from Charles River (France). C57BL/6 Tox −/− 10 were kindly provided by J. Kaye and crossed with C57BL/6J P14 TCR transgenic mice with a different CD45 allele to perform adoptive transfer experiments. C57BL/6J MOG-GP (MOGiCre/+ : Stop-GPflox/+) mouse line was generated by crossing mice expressing the Cre-recombinase under the control of the oligodendrocyte-specific promoter (C57BL/6J MOGiCre 55; with C57BL/6J Stop-GP mice 9). All mice were lodged under specific-pathogen-free P2 conditions in the animal facilities of the University Medical Center of Geneva. Male and female sex and age-matched mice between six weeks and twelve weeks of age were used for experiments. Mice were housed at 21°C and 50% humidity and bred under specific pathogen free conditions. Mice were exposed to a 12:12h light-dark cycle with unrestricted access to water and food.
Wild animals	No wild animals were used in this study.
Field-collected samples	No field-collected samples were used in this study.
Ethics oversight	All animal experiments were authorized by the cantonal veterinary office of Geneva and performed in agreement with the Swiss law for animal protection.

Note that full information on the approval of the study protocol must also be provided in the manuscript.

Flow Cytometry

Plots

Confirm that:

☐ The axis labels state the marker and fluorochrome used (e.g. CD4-FITC).

☒ The axis scales are clearly visible. Include numbers along axes only for bottom left plot of group (a 'group' is an analysis of identical markers).

☒ All plots are contour plots with outliers or pseudocolor plots.

☒ A numerical value for number of cells or percentage (with statistics) is provided.

Methodology

Sample preparation	Peripheral blood samples were obtained by facial vein puncture in heparin. Blood erythrocytes were lysed and cells fixed using BD FACS Lysing Solution. For the preparation of CNS-infiltrating leukocytes, mice were anesthetized and transcardially perfused with PBS. Brains were minced, digested in DMEM with Collagenase A (1mg/ml, Roche) and DNaseI (0.1 mg/ml, Roche) for 1h at 37°C and homogenized using 70-µm cell strainers (BD). Leukocytes were separated using a discontinuous percoll gradient (30 / 70%). Remaining erythrocytes were lysed using RBC Lysis buffer (Biolegend) for 3 min at RT. Surface staining was carried out with directly labeled antibodies in FACS buffer (2.5% FCS, 10 mM EDTA, 0.01% NaN3 in PBS). Isolated cells were quantified using AccuCheck Counting Beads (Invitrogen). Intracellular staining of TCF-1 and TOX was performed using FoxP3/Transcription Factor Staining Buffer Set (eBioscience) according to manufacturer's instructions. For ex vivo staining of Granzyme B, cells were fixed and permeabilized using commercial permabilization buffer set (Biolegend). To assess degranulation and intracellular cytokine production, brain leukocytes were cultured for 5h in the presence of 5 µg/ml FITC labeled anti-CD107a antibody, monensin (Biolegend) and brefeldin (Biolegend). Cells were stimulated in vitro with 1 µM KAVYNFATC peptide. Cells were fixed and permeabilized using commercial fixation/permabilization buffer set (Biolegend) followed by intracellular staining for cytokines.
Instrument	Flow cytometric samples were acquired on BD LSRFortessa (BD Biosciences) and Attune NxT Acoustic Focusing Cytometer (Thermo Fisher) using appropriate filter sets and compensation controls. In experiments which required high purity, P14 cells were sorted using Aria II flow cytometer within a laminar flow hood (BD Biosciences). For adoptive transfer experiment, naïve P14 cells from spleen were separated by AutoMACS (Miltenyi Biotec).
Software	Flow cytometry data were collected using BD FACSDiva (BD Biosciences v8.0.1) and Attune NxT Acoustic Focusing Cytometer software (Thermo Fisher, V3.1.2) and analyzed using FlowJo software (Treestar, V10).
Cell population abundance	Purity of P14 cells sorted by FACS was typically >99% and MultiMACS was typically >95% as confirmed by flow cytometry.
Gating strategy	For flow cytometric analysis the following gating strategy was used. First, leukocytes were gated on SSC-A/FSC-A, second, doublets were excluded with FSC-H/FSC-A, and third, dead cells removed (Near IR/FSC-A) .<br>For analysis of congenically marked P14 cells, cells were gated for CD8 and/or for the congenic marker.<br>For intracellular cytokine staining and transcription factors, cells were gated first on CD8 and CD45.1/CD45.2, and subsequently for the cytokines or transcription factors.

☒ Tick this box to confirm that a figure exemplifying the gating strategy is provided in the Supplementary Information.
